# Variants of *Epas1* contribute to hypoxia adaptation in the subterranean rodents *Eospalax* and *Spalax*

**DOI:** 10.26508/lsa.202603622

**Published:** 2026-07-20

**Authors:** Yibin Cao, Mengchen Zhang, Xin Li, Tianfu Yu, Yi Li, Hua Wang, Mengqi Liu, Xiaokang Xu, Fangyuan Xia, Kexin Li, Qiang Chen, Honghao Yu, Dengbang Wei, Yang Zhao, Eviatar Nevo, Jizeng Du, Xuequn Chen

**Affiliations:** 1 College of Chemistry and Life Science, Zhejiang Normal University , Jinhua, China; 2 NHC and CAMS Key Laboratory of Medical Neurobiology, Zhejiang University School of Medicine, Hangzhou, China; 3 Department of Neurology of the Second Affiliated Hospital, School of Brain Sciences and Brain Medicine, Zhejiang University, Hangzhou, China; 4 State Key Laboratory of Grassland Agro-Ecosystems and College of Ecology, Lanzhou University, Lanzhou, China; 5 School of Life Sciences, Lanzhou University, Lanzhou, China; 6 College of Biotechnology, Guilin Medical University, Guilin, China; 7 Research Center for High Altitude Medicine, Qinghai University, Xining, China; 8 Department of Physiology and Department of Hepatobiliary and Pancreatic Surgery of the First Affiliated Hospital, Zhejiang University School of Medicine, Hangzhou, China; 9 Institute of Evolution and International Graduate Center of Evolution, University of Haifa, Haifa, Israel

## Abstract

Oxygen-sensing and the hypoxia stress response play vital roles in physiological homeostasis. Subterranean species that are naturally adapted to hypoxia provide powerful tools for understanding the mechanisms of hypoxia tolerance. The plateau zokor *Eospalax baileyi* and the Gansu zokor *Eospalax cansus,* which inhabit the Qinghai–Tibet Plateau of China, and the blind mole rat *Spalax galili,* which lives in Israel, are all subterranean rodents adapted to hypoxic environments. Here, we showed that the T480S variant of the hypoxia-inducible factor-2ɑ (encoded by the endothelial PAS domain protein 1 gene, *Epas1*) in Chinese zokors stabilized EPAS1 by reducing phosphorylation, thereby regulating fibrosis and contributing to hypoxia adaptation on the Qinghai–Tibet Plateau. In Israeli blind mole rats, variants at positions -2023/-1810 in the *Epas1* regulatory region distinguish two abutting populations undergoing incipient sympatric speciation by altering the binding of cMYB and hepatocyte nuclear factor 4 gamma, linking transcriptional activity to the interferon mRNA transcription. This finding reveals an adaptive regulation of metabolism and O_2_ homeostasis by the *Epas1* gene in adaptation to diverse habitats, providing evidence for both divergent and convergent functional molecular evolution.

## Abstract (precis)

Subterranean rodents from China and Israel reveal convergent EPAS1 adaptation to hypoxia environments. Chinese zokors harbor a stabilizing T480S variant, whereas blind mole rats in Israel show regulatory variants linking transcription to interferons, illustrating divergent and convergent molecular evolution.

## Introduction

Subterranean rodents provide excellent models in which to understand the evolutionary adaptation to hypoxia. The blind mole rat *Spalax* lives in a complex subterranean tunnel system, inhabited by solitary individuals and characterized by fluctuating O_2_ levels, especially during winter floods (Nevo, 1961). In field measurements during the rainy season, O_2_ levels in the burrows have been detected at 7%, and CO_2_ levels at ∼6% (Shams et al, 2005). Remarkably, *Spalax galili* (*S. galili* 2n = 52), located in Eastern Upper Galilee, is one of the four subterranean blind mole rat species of the *Spalax ehrenbergi* superspecies in Israel, and underwent sympatric speciation ∼228,000 years ago, when one population migrated across a microscale from the original chalk, mildly hypoxic habitat, to a new abutting severely hypoxic volcanic basalt habitat (Nevo et al, 2000). This sympatric speciation was discovered by multidisciplinary comparison of the two populations by mitochondrial DNA (Hadid et al, 2013), genomic DNA (Li et al, 2015), transcriptome RNA-seq (Li et al, 2016), epigenomics (Zhao et al, 2016), and ecological–behavioral analyses (Lovy et al, 2015, 2017, 2020; Skliba et al, 2016). The O_2_ concentration is lower, and the CO_2_ concentration is higher in the heavy, clay basalt soil than in the relatively light chalk soil (Shams et al, 2005). Therefore, it is an ideal model in which to study the adaptive evolution of genes across a microscale soil divergence (Hadid et al, 2013). *Eospalax baileyi* and *Eospalax cansus* are closely related species of subterranean zokors in China; they inhabit the Qinghai–Tibet Plateau at elevations of 2,800–4,200 m (*E. baileyi*) and the Loess Plateau at elevations of 800–2,800 m (*E. cansus*). These animals spend most of their lives in sealed underground tunnels 1.5–2 m deep with no external aboveground access (Zhou & Dou, 1990), suffering hypoxia and hypercapnia. Particularly, 17.04–18.43% O_2_ and 0.22–1.46% CO_2_ (local ambient atmospheric values: 20.45% O_2_ and 0.03% CO_2_) have been measured in the burrows of *E. baileyi* (Zeng et al, 1984). *E. baileyi* and *E. cansus* have special adaptive mechanisms to the hypoxic–hypercapnic underground environment. For example, *E. baileyi* has higher red blood corpuscle counts and hemoglobin concentrations, but the hematocrit and mean corpuscular volume are lower than in other rodents (Wei et al, 2006).

Hypoxia-inducible factors (HIFs) play a central role in cellular responses to hypoxic conditions. HIFs are heterodimers of O_2_-regulated α-subunits (HIF-1ɑ and HIF-2ɑ) and a constitutively expressed β-subunit (HIF-β) (Semenza, 2011; Lee et al, 2020). HIF-1ɑ is ubiquitously expressed under stress and responsible for the regulation of a wide range of cellular hypoxic adaptive responses, and preferentially targets metabolic enzymes. A second isoform (EPAS1), encoded by the *Epas1* gene, shares sequence similarity with HIF-1ɑ. EPAS1 promotes adaptive responses to hypoxia and mediates angiogenesis and erythropoiesis (Keith et al, 2012; Befani et al, 2013; Lee et al, 2020). Moreover, the regulation of HIF stability, transcriptional activity, and cellular localization at the posttranslational level, like O_2_-dependent hydroxylation, ubiquitination, acetylation, and phosphorylation, has been investigated in numerous studies (Kietzmann et al, 2016; Taylor & Scholz, 2022).

Animals have developed genetic and epigenetic adaptations that permit their survival in extremely hypoxic plateau environments. Gene–environment interplay occurred on a millennial evolutionary timescale, and environmental adaptations are imprinted in genetic changes (Boyce et al, 2020). Single-nucleotide polymorphisms (SNPs) in the regulatory promoter, certain introns, and downstream of the *Epas1* gene are associated with the genetic adaptation to high-altitude hypoxia in Tibetans (Beall et al, 2010; Yi et al, 2010; Xu et al, 2014; Lou et al, 2015), Angus cattle (Newman et al, 2015), the Tibetan gray wolf (Zhang et al, 2014), and the zokor (*E. baileyi*) (Cai et al, 2018). Metabolic responses to hypoxia include HIF-mediated reprogramming of the metabolism toward aerobic glycolysis, and a decrease in fatty acid oxidation (O'Brien et al, 2020).

Acute hypoxia and chronic hypoxia have distinct effects on tissue fibrosis. For instance, transient hypoxia may promote the repair process during normal tissue repair processes, whereas chronic hypoxia can lead to excessive scar formation because of driving the process of fibrosis and impair organ function (Darby & Hewitson, 2016). In the process of renal fibrosis, transient hypoxic preconditioning before kidney injury can have a protective effect, whereas chronic hypoxia may cause severe damage to renal function (Naas et al, 2023). In this study, we report that a T480S variant in the coding DNA sequence (CDS) inhibits the phosphorylation of EPAS1 in *E. baileyi* and *E. cansus* in China, and this results in the accumulation of EPAS1. In parallel lines of investigation, four SNPs in the *Epas1* regulatory region were found in two adjacent populations of *S. galili* in Israel, and these SNPs alter *Epas1* gene transcription. These variants resulted in suppression of the interferon signaling pathway and fibrosis in *S. galili* basalt population, *E. baileyi* and *E. cansus*, which may contribute to the ecological adaptation of these species to their subterranean environments.

## Results

### Sequence variants in the regulatory region of *S. galili Epas1* and the CDS of *E. baileyi* and *E. cansus Epas1*

**Figure S1.**
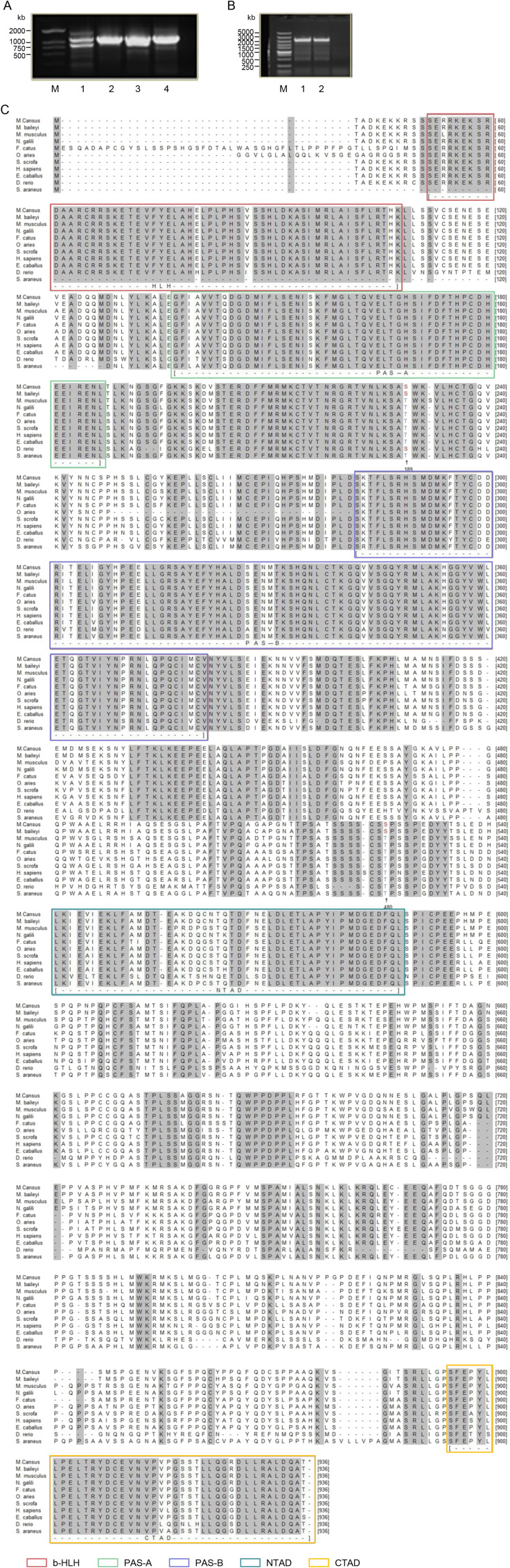
Cloning and sequence analysis of *Epas1* from *E*. baileyi and E. cansus. **(A)** Cloning of the 1.2-kb fragment of the *S. galili Epas1* promoter. M: DL 2000 DNA Marker: 1–4: samples B1, B2, C1, and C2. **(B)** Cloning of the 3-kb fragment of the *S. galili Epas1* promoter. M: DL 5000 DNA Marker: 1. sample B1; 2. sample C1. **(C)** Alignment of amino acid sequences of EPAS1“*Myospalax baileyi *(*M. baileyi*), *Myospalax cansus *(*M. cansus*), *Sorex araneus *(*S. araneus*)”. of *E. baileyi(=M. baileyi)* and *E. cansus (M. cansus)* with mouse (*Mus musculus*, AAB41496.1), blind mole rat (*S. galili*, XP_008821290.1), cat (*Felis catus*, XP_003984034.1), sheep (*Ovis aries*, XP_004007338.1), pig (*Sus scrofa*, NP_001090889.1), human (*Homo sapiens*, NP_001421.2), horse (*Equus caballus*, XP_005600062.1), European shrew (*S. araneus*, XM_004612047), and zebrafish (*Danio rerio*, NP_001034895.2). Nucleotide sequence of *E. baileyi* EPAS1 cDNA and the deduced protein sequence (GenBank accession No. MF461621).

**Figure 1.**
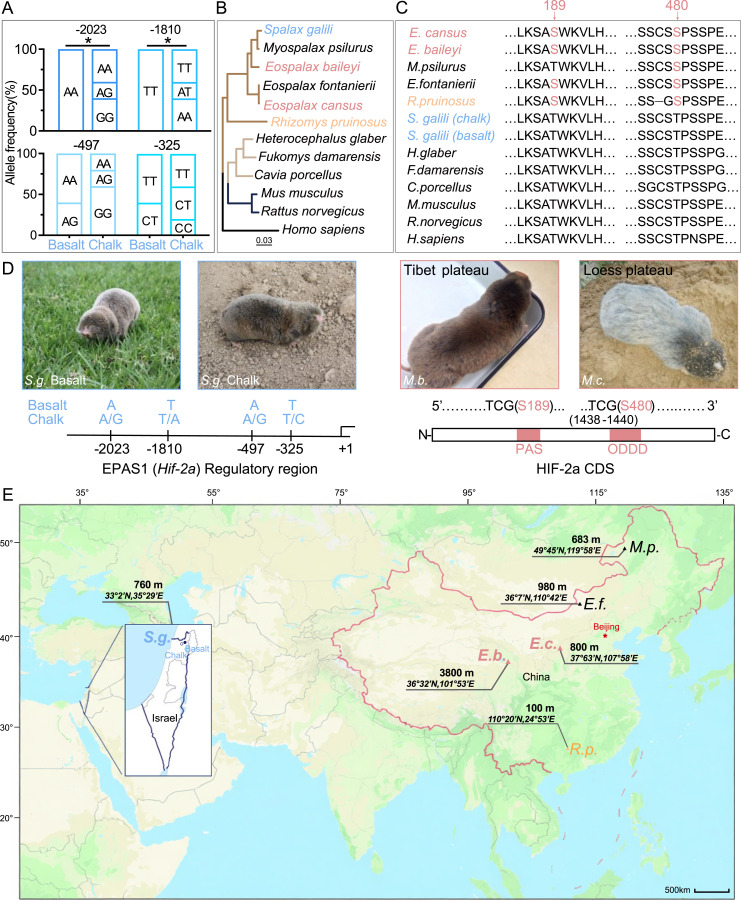
Four variants in the *Epas1* regulatory region of *S. galili* chalk and *S. galili* basalt in Israel, and two variants (S189 and S480) in the *Epas1* CDS of *E. baileyi* and *E. cansus* in China. **(A)** Four variants at the -2023, -1810, -497, and -325 sites of the *Epas1* regulatory region of *S. galili* basalt and *S. galili* chalk in Israel. *P < 0.05 by Fisher’s exact test. **(B)** Phylogenetic tree for EPAS1 constructed using a matrix based on protein sequences and the program Mega 6. **(C, D)** S480 in EPAS1 of *E. baileyi* and *E. cansus* substitutes the conserved Thr residue in EPAS1 of other vertebrates. Our laboratory members and authors (KX Li. for *S. galili*) took animal photographs during field observations in the local area. **(E)** Geographical distribution of underground mole rats in Israel and China (imagery and map data ©2019 Google, TMap Mobility, Mapa GISrael). CDS, coding DNA sequence; ODDD, O2-dependent degradation domain.

The upstream regulatory regions of *Epas1* from *S. galili* basalt and *S. galili* chalk in Israel were cloned by nested PCR. Sequence comparison of these populations revealed four variants in the 4-kb upstream regulatory region of the *Epas1* gene (Fig S1A and B). The SNPs were diverse in chalk mole rat populations and relatively conserved in basalt populations. The frequencies of the -325 T and -497 G alleles in the *S. galili* chalk population were higher than those in the *S. galili* basalt population (although the difference was not significant). Moreover, the A allele was overrepresented in the basalt population compared with G in the chalk population at the -2023 site, and the T allele was overrepresented in the basalt population compared with A in the chalk population at the -1810 site (*P* = 0.032 by Fisher’s exact test, Fig 1A). The allele frequency differences between the basalt and chalk populations of *S. galili* are based on 10 individuals each. Although we are not claiming the variants to be population-diagnostic, the -2023 site showed significant differentiation between basalt and chalk populations, with the AA genotype at 0.4 in chalk population and fixed (1.00) in basalt population. The same tendency was found in the -1810 site, with the TT genotype being 0.4 in chalk population and fixed (1.00) in basalt population. Larger sample size would strengthen the statistical power of the variation, but collecting additional individuals from the wild has proven to be logistically challenging. To assess the potential link between the regulatory sequence variants to hypoxia fitness differences of the two populations, Electrophoretic mobility shift assay (EMSA), luciferase reporter assay, and RNA-seq were used to evaluate and clarify the functional relevance.

Although no SNPs were identified in the regulatory regions of HIF-2ɑ either of *E. baileyi* or of *E. cansus* in China, their CDS varied. The CDS of Epas1 from *E. baileyi* and *E. cansus* in China coded proteins of 868 aa and 871 aa, respectively (GenBank accession Nos. MF461621 and MK085980). The sequence identity of *E. baileyi* HIF-2ɑ with *E. cansus*, *S. galili*, and *Rhizomys pruinosus* was 97.9%, 96.1%, and 94.7%. *E. baileyi* in China had identities of 59.5%, 88.2%, and 89.7% with rat, mouse, and human HIF-2ɑ protein, respectively (Fig 1B). Evidence implies that the interval between the separation of *Myospalacinae* and *Rhizomyinae* and the initial speciation preceding the divergence of *Spalacidae* is relatively short (Lin et al, 2014). Approximately 29.1 million years ago (Mya), blind mole rats diverged from zokors, followed by the divergence between zokors and bamboo rats around 26.9 Mya (Guo et al, 2020). There were two variants of T189S and T480S from *E. baileyi*, *E. cansus*, and *R. pruinosus* in China, which were not found in *S. galili* in Israel (Fig 1C–E). The T480S of *E. baileyi* EPAS1 and *E. cansus-*EPAS1 is a unique variant in the conserved dipeptide motif T480:P481 in the O_2_-dependent degradation domain of the EPAS1 protein, which is not variant among non-*Spalacidae* mammals, and S189T is also conserved in other mammals (Fig S1C). In the M2 and M8 model of the branch test, the Ala at position 27 (according to *E. baileyi* EPAS1) had posterior probabilities of 0.979 and 0.997 as indicated by the Bayes–empirical Bayes method, which were significant at the 5% and 1% level, respectively (Table S1). But in the branch-site test, no significantly positive selection was detected along the lineages leading to *E. baileyi* and *E. cansus* (Table S2).

Table S1. Likelihood-ratio test (LRT) of positive selection in the *Epas1* gene for vertebrates.

Table S2. Branch-site test of positive selection (branch-site model A, test 2) in the *Epas1* gene of *Eospalax*, *Spalax*, and naked carp.

### The mRNA levels of *Epas1* and its target genes are changed in the liver and lung of *S. galili*, *E. baileyi*, and *E. cansus*

**Figure 2.**
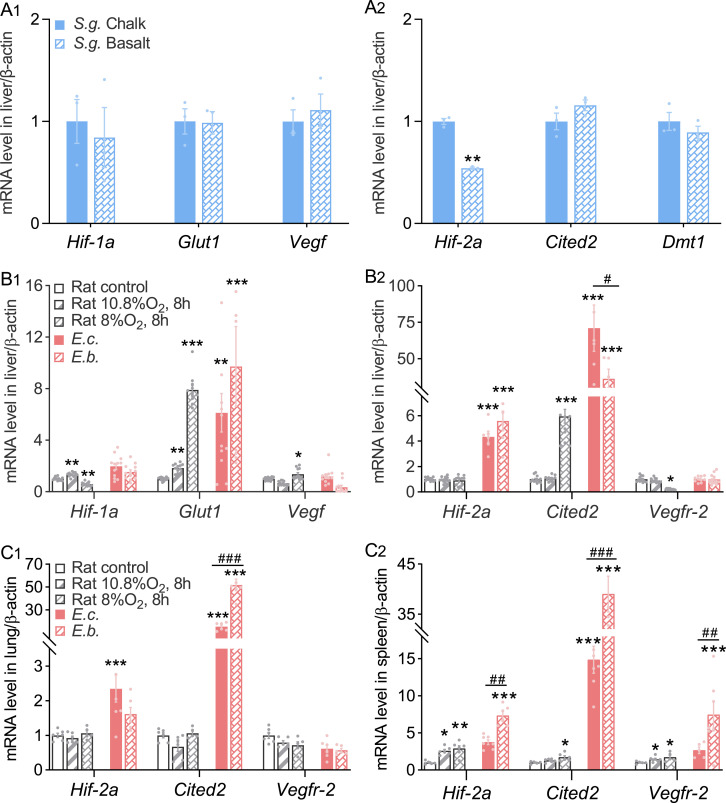
Comparison of mRNA levels of Hif-1a, *Epas1*, and target genes in the tissues of laboratory rats, S. galili, *E. baileyi*, and E. cansus. **(A**_**1**_**)** *Hif-1a* and target gene *Glut1*, *Vegf* mRNA expression in the liver of *S. galili* basalt and *S. galili* chalk. **(A**_**2**_**)** *Epas1* and target gene *Cited2* and *Dmt1* expression in the liver. **(B**_**1**_**)** *Hif-1a* and target gene expression in the liver of laboratory rats under hypoxia, *E. baileyi*, and *E. cansus*. **(B**_**2**_**)** *Epas1* and target gene expression in the liver. **(C**_**1**_**)** mRNA expressions of *Epas1* and target genes in the lung of laboratory rats under hypoxia, *E. baileyi*, and *E. cansus*. **(C**_**2**_**)** mRNA expressions of *Epas1* and target genes in the spleen. Data are expressed as the mean ± SEM, with each point representing one replicate mean. Statistical comparisons in different treatments were performed using a one-way ANOVA and paired *t* test. *, ^#^*P* < 0.05, ^##^, **P < 0.01, ***, ^###^*P* < 0.001 versus rat control or *S. galili* chalk, n = 6.

The expression of *Hif-1a*, *Epas1*, and their target genes in the tissues of *S. galili*, *E. baileyi*, and *E. cansus* was analyzed by qRT-PCR. The expression level of *Epas1* in hepatic tissue in the basalt population was significantly lower than that in the chalk population. Apart from that, no significant change in *Hif-1a* and *Epas1* target genes (*Glut1* and *Vegf*, *Cited2* and *Dmt1*) was found in the liver (Fig 2A_1_ and A_2_). The mRNA levels of *Hif-1a* and *Vegf* in the liver of zokors showed no significant differences compared with those of rats, but *Glut1* mRNA was up-regulated (Fig 2B_1_). The mRNA levels of *Cited2*, the HIF-2 target gene that inhibits the hypoxic induction of *Vegf* in both Hep3B and HEK293 cells (Shin et al, 2008), were significantly higher in liver (Fig 2B_2_), lung (Fig 2C_1_), and spleen (Fig 2C_2_) tissues of *E. baileyi* and *E. cansus* than in hypoxia-treated laboratory rats. *Vegfr-2* mRNA was higher in *E. baileyi* spleen, but decreased in the liver and increased in the spleen under hypoxia in laboratory rats (Fig 2B_2_, C_1_, and C_2_).

### Variants mediate the binding of specific transcription factors in the *S. galili Epas1* regulatory region and regulate the promoter activity of the *S. galili Epas1* gene

**Figure 3.**
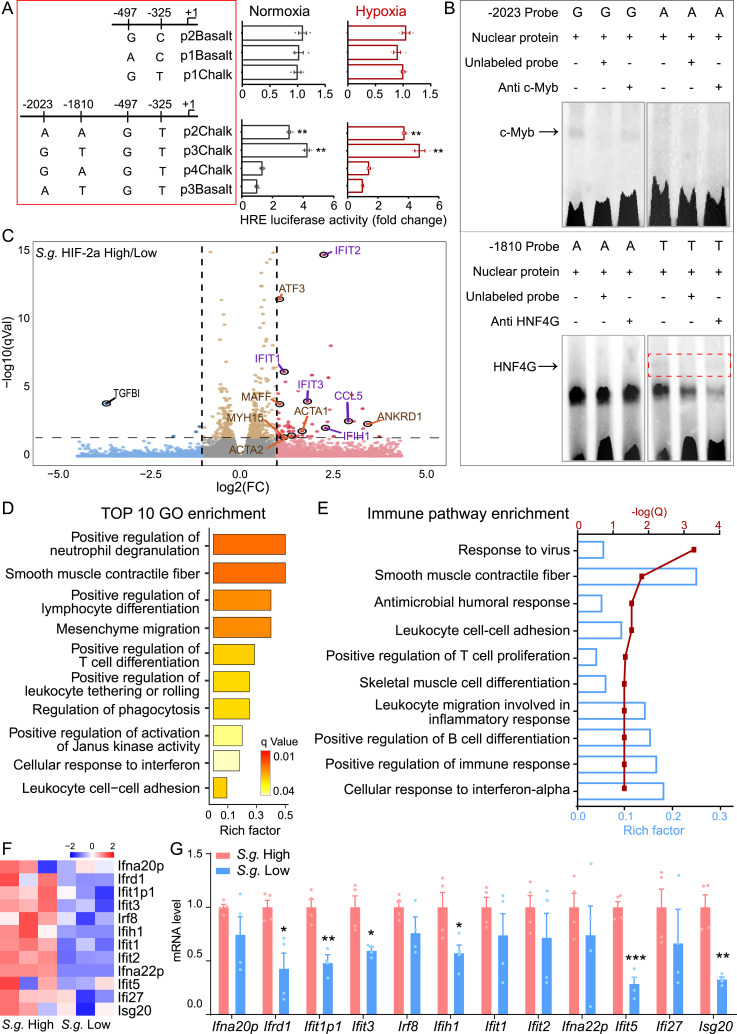
Variants in the regulatory region alter the promoter activity and functions of *S. galili* EPAS1. **(A)** *Epas1* variants in *S. galili* basalt showed the lowest promoter activity under normoxia and hypoxia. **(B)** -2023A variant of *S. galili* *Epas1* decreased the transcription binding affinity of cMYB, and the -1810T variation provides the binding site for HNF4G in the EMSA test. **(C, D, E, F)** Transcriptome analysis of high-dose and low-dose *S. galili* EPAS1. The DEGs were shown by volcano plots (C), DEGs were enriched in immune pathways (D, E), and interferon-related DEGs were shown in heatmaps (F). **(G)** qRT-PCR analysis of interferon-related genes in the high dose or low dose of *S. galili* EPAS1 in HEK293T cells. Data are the mean ± SEM, with each point representing one replicate mean. Statistical comparisons in different groups were performed using a one-way ANOVA and paired *t* test. *P < 0.05, **P < 0.01, ***P < 0.001 versus p3Basalt or *S. galili* EPAS1 high dose, n = 3. p1-3Basalt, reporter plasmids with basalt version; p1-4Chalk, reporter plasmids with chalk version.

**Figure S2.**
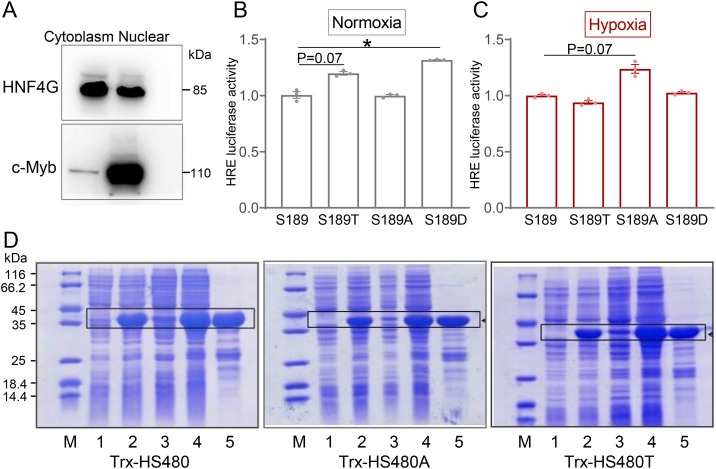
Expression and transcriptional activity of EPAS1 and regulators. **(A)** Protein expression of HNF4G and cMYB in HeLa cells. **(B, C)** *E. baileyi* EPAS1 transcriptional activity with 189T, 189A, 189S, and 189D under normoxia and hypoxia. Values are shown as the mean ± SEM, with each dot representing one replicate mean. Statistical comparisons among treatments were performed using a one-way ANOVA and paired *t* test. n = 3. *P < 0.05 versus S189 group. **(D)** Prokaryotic expression of Trx-HS480, Trx-HS480A, and Trx-HS480T.

To quantify the impact of regulatory variants, we measured the promoter activities of Epas1 on a reporter gene using dual-luciferase reporter assays. Although the -325 T>C and -496 G>A did not affect *S. galili Epas1* promoter activity, both -2023G>A and -1810A>T, which are significantly more frequent in *S. galili* basalt, reduced the promoter activity, with the combination of -2023A and -1810T showing the lowest activity (Fig 3A). Bioinformatics analysis predicted that -1810 A>T and -2023 G>A SNPs may influence the recognition and binding of cMYB and HNF4G. cMYB acts as a transcriptional activator, and the heterodimer of HNF4G and HNF4a reduces transcriptional activity. EMSA was performed using wild- and variant-type biotin-labeled double-stranded probes. We found that -2023A abolished the binding of cMYB in the upstream regulatory region of *Epas1*, whereas -1810T increased the binding of HNF4G at the *Epas1* regulatory region (Figs 3B and S2A).

We applied GO and KEGG enrichment analyses after transfection with either high-dose or low-dose EPAS1 plasmids, which were used to mimic the two distinct *S. galili* subgroups. The outcomes of GO analyses showed that differential gene expressions (DEGs) were most significantly enriched in interferon-related genes, and immune pathways such as smooth muscle contractile fiber, differentiation of immune cells, and cellular response to interferon (Fig 3C–E). The interferon-related genes were significantly decreased in *S. galili* EPAS1 low (Fig 3F and G), which indicates that the low levels of EPAS1 mediate down-regulation in the immune function of *S. galili* basalt.

### Variants increase *E. baileyi* EPAS1 protein stability through down-regulating phosphorylation

**Figure 4.**
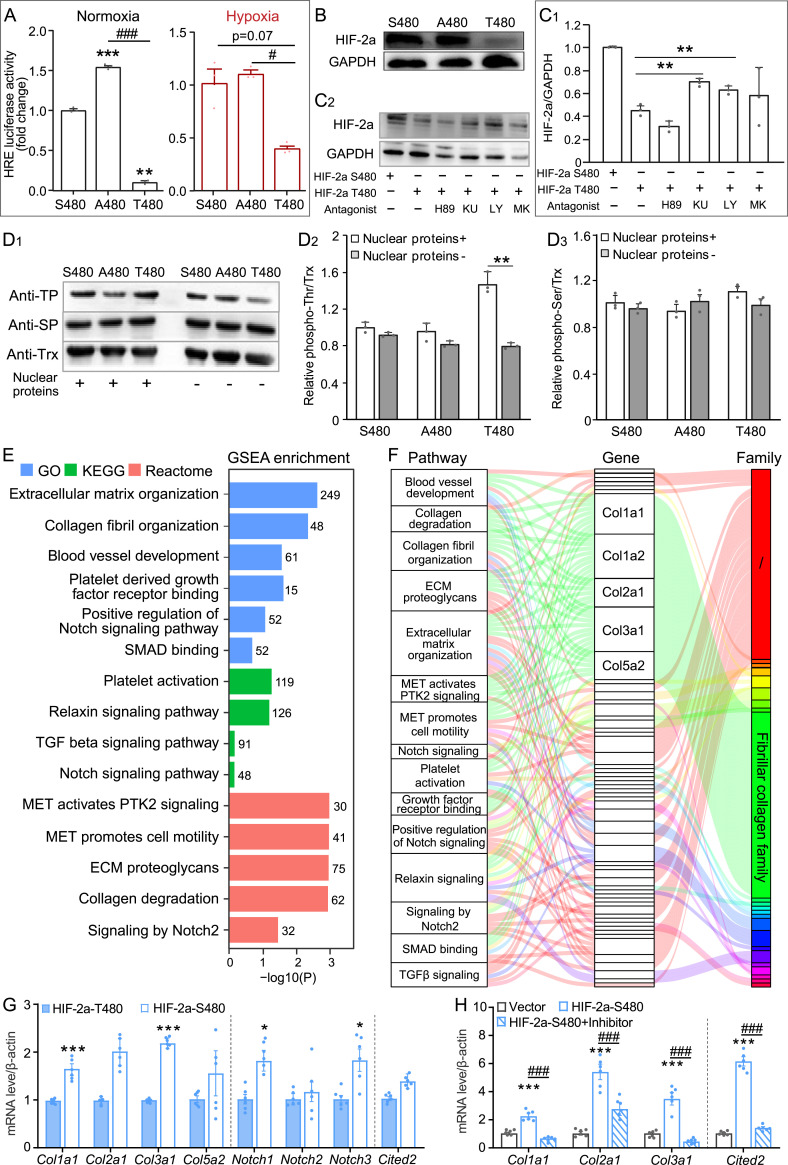
Variant of *E. baileyi *EPAS1 mediates the phosphorylation and collagen-related pathways. **(A)** Transcriptional activity of T480 in *E. baileyi* EPAS1 is decreased under normoxia and hypoxia. **(B)** EPAS1 S480 showed higher protein levels. **(C**_**1**_**, C**_**2**_**)** Identification of the signal pathways for the phosphorylation of EPAS1, H89: PKA inhibitor; KU: ATM kinase inhibitor; LY: Raf kinase inhibitor; MK: AKT inhibitor. **(D**_**1**_**, D**_**2**_**, D**_**3**_**)** T480 presents a stronger phosphorylation signal than those of A480 and S480 in vitro. **(E, F)** ECM pathways and collagen family genes were enriched in the *E. baileyi* EPAS1 S480 and T480 transcriptomes. **(G)** EPAS1-S480 significantly induced the mRNA expression levels of *Col1a1*, *Col3a1*, *Notch1*, and *Notch3* genes compared with the transfection with EPAS1-T480 in HEK293T cells. **(H)** EPAS1 inhibitor PT2977 suppressed the EPAS1-S480–induced *Col1a1*, *Col2a1*, *Col3a1* expression. Data are presented as the mean ± SEM, with each point representing one replicate mean. Statistical comparisons among treatments were performed using a one-way ANOVA and paired *t* test. *, ^#^*P* < 0.05, **P < 0.01, ***, ^###^*P* < 0.001 versus S480, T480, or vector, n = 3.

**Figure S3.**
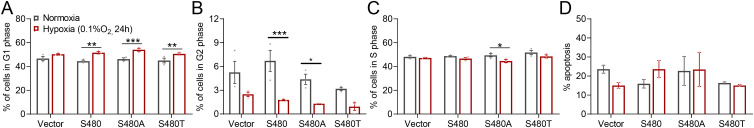
Variation of *E. baileyi EPAS1* S480-induced cell-cycle arrest and apoptosis under normoxia and hypoxia. **(A, B, C)** Variations of S480 increase the percentage of G1 and decrease G2 in hypoxia. **(D)** Percentage of apoptosis under normoxia and hypoxia. Values are presented as the mean ± SEM, with each point representing one replicate mean. Statistical comparisons among treatments were performed using a one-way ANOVA and paired *t* test. n = 3, *P < 0.05, **P < 0.01, and ***P < 0.001 versus normoxia group.

To investigate the potential posttranslational modification and possible functional alterations of the *E. baileyi* EPAS1 protein induced by variants within the CDS in *E. baileyi*, we transiently transfected HeLa cells with various combinations of plasmids of pEGFP-EPAS1 (S480), pEGFP-EPAS1 (A480), and pEGFP-EPAS1 (T480) to validate the change of EPAS1 protein levels. For Chinese zokors with variants of 189 and 480 in the CDS of *Epas1*, transcriptional activity toward a hypoxia response element luciferase reporter was assessed. *E. baileyi* EPAS1 (T480) showed a ∼70% decrease in transcriptional activity than its S480 or A480 variants under normoxic and hypoxic conditions (Fig 4A), and the variants of 189T, 189A, 189S, and 189D had no significant impact on the transcriptional activity of *E. baileyi* HIF-2 (Fig S2B and C). Markedly, there was a decrease in the EPAS1 protein level in pEGFP-EPAS1 (T480)–transfected cells (Fig 4B). Furthermore, four kinase inhibitors, PKA inhibitor (H89), ataxia-telangiectasia mutated (ATM) kinase inhibitor (KU), Raf kinase inhibitor (LY), and AKT inhibitor (MK), were applied to identify the kinase pathway involved in the phosphorylation of 480. The T480-induced repression of the EPAS1 protein level was partially blocked by ATM kinase inhibitor and Raf kinase inhibitor, suggesting a possible role of ATM and the Raf kinase pathway in the T480 phosphorylation of EPAS1 (Fig 4C_1_ and C_2_). MAPKs are proline-directed serine/threonine kinases, which phosphorylate the serine/threonine in the dipeptide motif S/TP; thus, the T480S variation may influence the phosphorylation of *E. baileyi* EPAS1. To confirm that T480S can regulate the phosphorylation of *E. baileyi* EPAS1, we cloned the 420–540 fragment of *E. baileyi* EPAS1 into the pET-32a vector, and the S480 residue was mutated to Ala or Thr by site-directed mutagenesis (Fig S2D). Because T480 is followed by a proline residue, we tested whether this site reacts with an anti-phospho-Thr/Ser-Pro antibody. As expected, nucleoproteins were very efficient in phosphorylating *E. baileyi* HIF-2 at T480. Substitutions of T480 with S480 or A480 residues caused dramatic decreases in the phosphorylation signal. In contrast, there was no change in the phosphorylation level after using a phospho-Ser-Pro antibody (Fig 4D), suggesting that T480 is a major target of phosphorylation. EPAS1 has been reported to stabilize cMYB-MAX complexes, thereby increasing the transcription of cMYB target genes required for cell proliferation (Gordan et al, 2007; Semenza, 2011). In this study, S480, A480, and T480 induced G1-phase arrest under hypoxia. Meanwhile, S480 and A480 decreased the percentage of G2-phase cells, and A480 decreased S-phase cells under hypoxic conditions (Fig S3A–C), but did not change apoptosis (Fig S3D), suggesting that S480 may regulate the cell cycle *via* phosphorylation.

To investigate the functions of EPAS1 codon 480 variations, we transfected EPAS1-T480 or EPAS1-S480 plasmids into HEK293T cells, followed by transcriptomic analysis. The DEGs were enriched in extracellular matrix (ECM) organization, collagen degradation, the TGF-β and Notch2 signaling pathway, blood vessel development, and platelet activation. In addition, the pathway genes were enriched in genes of the fibrillar collagen family, including *Col1a1*, *Col1a2*, *Col2a1*, *Col3a1*, and *Col5a2* (Fig 4E and F). Compared with EPAS1-T480 (human and rat), the mRNA levels of *Col1a1*, *Col3a1*, *Notch1*, and *Notch3* were up-regulated in EPAS1-S480, and this could be abolished by the EPAS1 inhibitor PT2977 (Fig 4G and H).

### *E. baileyi* EPAS1 is involved in antifibrosis in primary lung fibroblasts

**Fig. 5.**
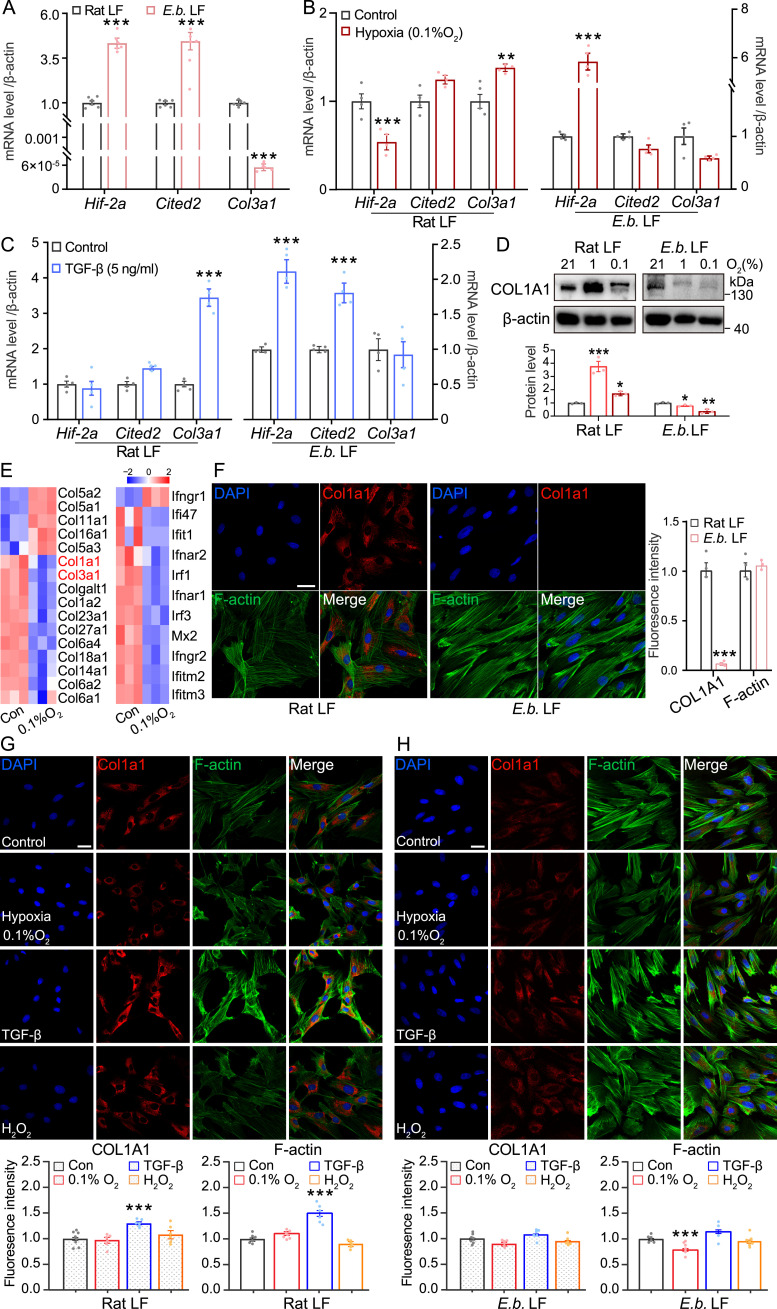
Plateau zokors showed antifibrosis in primary lung fibroblasts. **(A)** mRNA levels of *Epas1* and its target gene *Cited2* were significantly higher in *E. baileyi* LFs compared with rats under normoxia, whereas *Col3a1* was much lower in *E. baileyi* LFs. **(B)** Hypoxia up-regulated the mRNA expression of *Col3a1* in rat LFs, but only induced *Epas1* gene expression in *E. baileyi* LFs. **(C)** TGF-β treatment significantly induced an increase in *Col3a1* mRNA levels in rat LFs, but in *E. baileyi* LFs, *Epas1* and *Cited2* mRNA levels significantly increased, whereas *Col3a1* mRNA showed no significant change. **(D)** Western blot analysis revealed that hypoxia promoted COL1A1 protein expression in rat cells, whereas hypoxia inhibited COL1A1 protein expression in *E. baileyi* LFs. **(E)** mRNA expressions of collagen family genes and interferon-related genes were decreased in *E. baileyi* LFs under hypoxia. **(F)** COL1A1 protein showed much lower fluorescence intensity in *E. baileyi* LFs compared with rat cells in immunofluorescence analysis. **(G, H)** TGF-β treatment significantly induced fibrosis in primary rat LFs, but had no significant effect on *E. baileyi* LFs. Scale bar: 30 μm. Data are the mean ± SEM, with each point representing one replicate mean. Statistical comparisons among different treatments were performed using a one-way ANOVA and paired *t* test. *P < 0.05, **P < 0.01, ***P < 0.001 versus rat LF or control, n = 3.

Collagens are the main components of the ECM, and overaccumulation can induce fibrosis. We isolated the primary lung fibroblasts (LFs) from laboratory rats and *E. baileyi,* and surprisingly found that the basal mRNA levels of *Epas1* and *Cited2* in *E. baileyi* were indeed higher than those of rats under normoxic conditions; however, *Col3a1* was rarely expressed in *E. baileyi* LFs (Fig 5A). Hypoxia (0.1% O_2_) and TGF-β treatments increased the *Col3a1* mRNA in rat LFs, but not in *E. baileyi* LFs, whereas these treatments increased the *Epas1* mRNA in *E. baileyi* LFs, but decreased it in rat LFs (Fig 5B and C). COL1A1 is a key marker of lung fibrosis, and was significantly activated in rat LFs under 1% or 0.1% O_2_. In contrast, in plateau zokor fibroblasts, the protein levels of COL1A1 were lower than those of rat LFs, and decreased under both 1% and 0.1% O_2_ (Fig 5D). The down-regulated expression of collagen family genes and interferon-related genes was found in *E. baileyi* LFs under normoxic and hypoxic conditions (Fig 5E). The interferon pathway showed the same transcriptional regulation patterns in the plateau zokor and *S. galili* basalt population (Fig 3F), indicating that relatively insensitive immune functions might benefit adaptations to hypoxia in subterranean rodents (*E. baileyi* in China and *S. galili* in Israel). The basal COL1A1 fluorescence intensity was markedly lower in *E. baileyi* LFs versus rat LFs (Fig 5F). TGF-β treatment increased COL1A1 and F-actin (fibrosis markers) levels in rat LFs, but no change in *E. baileyi* LFs was found. Astonishingly, the F-actin fluorescence intensity was decreased in *E. baileyi* LF under hypoxia, which indicated that plateau zokor LFs have stronger resistance to hypoxia and TGF-β–induced fibrosis (Fig 5G and H).

## Discussion

HIF-2ɑ (*Epas1*) is a regulatory hub that plays a crucial role in response to hypoxic stress. Both *S. galili* chalk and basalt populations live in an underground habitat with fluctuating O_2_ and CO_2_: 17–21% O_2_ in the dry season and 7% O_2_ in the rainy season. In a previous study with a comprehensive epigenetic and functional analysis of TP53 in *S. galili* chalk and basalt, we found that different methylation modifications of p53 contribute to an adaptive shift in supervising its target genes (Zhao et al, 2016). Moreover, a variant of TP53 in *E. baileyi* and *E. cansus* from China occurs at codon 104 of the CDS (Zhao et al, 2013), whereas mutation of TP53 in *S. galili* chalk and basalt is at codon 172, which mimics a human mutation hot spot in cancer. Therefore, we hypothesize that variants in the regulatory region of *S. galili Epas*1 affect its transcription by altering the binding of certain transcription factors, which may be involved in responding to fluctuating O_2_ and CO_2_ levels in subterranean caves. Our results do not indicate that the variations directly drive the separation between the chalk and basalt populations, as there has been no evidence linking these variations to reproductive isolation and hence speciation. However, the regulatory outcome by these variations may benefit their adaptation.

In the present study, we revealed that the variants in the regulatory region of *S. galili Epas1* were located in two potential binding sites for cMYB and HNF4G. *Epas1* is activated by aMYB and cMYB (Lei et al, 2004), and the EPAS1 protein interacts with bMYB in the nucleus of 786-O cells (Okumura et al, 2017). MYB is recognized as a crucial hematopoietic stem cell regulator for red cell differentiation, as well as an enhancer regulating fetal hemoglobin (Wang et al, 2018; Shah et al, 2021). In the present study, the binding of cMYB to the regulatory region of *Epas1* may represent increased promoter activity and mediate hematopoiesis and erythropoiesis under hypoxia. Stronger repression of *Epas1*, *Hif-1a*, and *Hnf4* is correlated with a down-regulation of genes in the I, III, and V complexes of the electron transport chain, suggesting that the reduction in mitochondrial activity is compatible with HIF-1 system activation in the blood of a long-distance runner (Maqueda et al, 2017). Our results suggest that the variant in *S. galili* basalt (-1810T and -2023A) represses the promoter activity of *Epas1* through promoting the binding of HNF4G and abrogating the cMYB-binding site. Unexpectedly, the luciferase activity of a chalk regulatory region version (-2023G and -1810A) was also lower than the other two chalk regulatory region versions (-2023G and -1810T; -2023A and -1810A), perhaps because of the synergistic effects of the two transcriptional factors. Consistent with the results of qRT-PCR, the -1810 A>T and -2023 G>A variants significantly down-regulated the promoter activity of *Epas1* in the *S. galili* basalt population; consequently, no overexpression of HIF-2 was found. We realize that a ChIP-seq in native *Spalax* tissues would be the best proof of the mechanisms. However, ChIP-grade antibody against the *Spalax* EPAS1 protein is not currently available. We therefore performed our experiments in HEK293T cells to demonstrate the general regulatory mechanisms. Taken together, these data imply that the variants in *S. galili* basalt (-1810T and -2023A) are loss-of-function variants that directly affect the transcription of *Epas1* mRNA.

Loss-of-function variants of *Epas1* have been found to be enriched in the native Tibetan people (Beall et al, 2010; Yi et al, 2010; Xu et al, 2011) and Tibetan dogs (Gou et al, 2014), and may be associated with lower blood flow resistance, playing a beneficial (or detrimental) role under chronic hypoxia. *S. galili* has more red blood cells (RBCs), hemoglobin, and a higher hematocrit (HCT) than *Spalax carmeli*, *Spalax judaei*, and laboratory rats. It has been hypothesized that the genetic differences between the species have resulted from the relocation of *Spalax* ancestors to live underground (Danial-Farran et al, 2017). The higher RBCs, hemoglobin, and HCT in *S. galili* may be beneficial for adaptation to hypoxic underground life, especially during winter floods, but also increases the risk of polycythemia and vascular resistance. Our results showed that the -1810T and -2023A variants in *S. galili* basalt induced a decrease in EPAS1 mRNA levels, benefiting *S. galili* by avoiding the risk of erythrocytosis. In previous work, researchers did not distinguish the blood properties of the basalt population from those of the chalk population (Danial-Farran et al, 2017), suggesting that these variations may blunt the EPAS1-mediated erythropoiesis and protect the *S. galili* basalt population, which resides in hypoxic heavy soil, from erythropoiesis. Similarly, down-regulation of *Epas1* transcription has also been reported to be associated with the genetic adaptation to high-altitude hypoxia in Tibetans (Peng et al, 2017) and Tibetan pigs (Ma et al, 2019).

To further investigate whether the functional changes in *Epas1* provide other adaptive benefits for the subterranean rodent *S. galili*, we applied transcriptomic analyses to the high-dose group and the low-dose group of *S. galili* EPAS1 plasmid treatment in HEK293T cells. The GO enrichment analyses revealed the top 2 enrichment (Q <0.01) in the following categories: smooth muscle contractile fiber and positive regulation of neutrophil degranulation. It has been reported that living at high altitudes causes hypoxic pulmonary hypertension, which is a complication of many lung diseases, including chronic obstructive pulmonary disease and cystic fibrosis, and vascular collagen content is important to the extralobar pulmonary artery stiffening caused by chronic hypoxia (Boyce et al, 2020). Chan et al generated EPAS1 gain-of-function–induced pluripotent stem cells and differentiated them into endothelial cells (ECs) and smooth muscle cells (SMCs). EPAS1-SMCs, but not EPAS1-ECs, were phenotypically aberrant, more contractile, stiffer, and overexpressed endothelin 1, myosin heavy chain, elastin, and fibrillin (Keith et al, 2012). The hypoxia-induced enrichment of contractile fiber genes has been reported in the brain of *Spalax* (Malik et al, 2012). We hypothesized that the loss-of-function variant in the regulatory region of the *S. galili Epas1* gene not only inhibits erythrocytosis but also mitigates hypoxia-induced disorganized stress fibers and higher stiffness in their arterial SMC.

Variants of the *Epas1* gene associated with hypoxic adaptation are found not only in regulatory regions (introns, promoters, and 3′ downstream regions) in Tibetans (Beall et al, 2010; Yi et al, 2010; Hu et al, 2017) and other hypoxia-tolerant vertebrates (Zhang et al, 2014; Newman et al, 2015), but also in the coding regions of the gene, such as the Glu76Asp variant in the naked mole rat *Epas1* (Shao et al, 2015). This study examined the function of the S480T amino acid substitution in EPAS1, which may be one of the important strategies to adapt to extreme highland environments and the burrow microenvironment of *E. baileyi* and *E. cansus* The transcription factors can be regulated by phosphorylation either positively or negatively to maintain complex homeostasis. Numerous kinases regulate the phosphorylation of HIF-1ɑ and EPAS1 in HIF stability, subcellular localization, and transactivity (Kietzmann et al, 2016). We identified the S189 and S480 variants at the PAS and O_2_-dependent degradation domain of EPAS1 in *E. baileyi* and *E. cansus* T480S of EPAS1 are unique to *E. baileyi* and *E. cansus* in China compared with all other vertebrates. This variant abolishes phosphorylation, resulting in enhanced EPAS1 (S480) transcriptional activity and displaying a potential gain-of-function. However, without direct measurements of EPAS1 protein half-life or degradation kinetics, the available information is insufficient to explain the stability of the mutated EPAS1.

Gain-of-function mutations of HIF-2 are known to cause erythrocytosis, resulting in an increased number of RBCs through their regulation of erythropoietin in humans (Gale et al, 2008) and high-altitude goat populations (Song et al, 2016). Cattle at high altitudes also have similar gain-of-function variants in exon 12 associated with pulmonary hypertension (Newman et al, 2015). Although these gain-of-function mutations are largely considered deleterious in both humans and cattle, there is a lower HCT and mean corpuscular volume without erythrocytosis in hemolysis tests from subterranean *E. baileyi* and *E. cansus* (versus lowland laboratory rats) (Wei et al, 2006). One possible explanation for the resistance to erythrocytosis in *E. baileyi* and/or *E. cansus* is the up-regulated transcription of *Cited2*. *Cited2* is preferentially up-regulated by EPAS1 and exhibits a high affinity for the Zn-binding cysteine/histidine-rich 1 domain of the transcriptional coactivators cAMP response element-binding protein/p300, for which it competes with HIFs (Fernandes et al, 2020; Usui-Ouchi et al, 2020), and the hypoxia-induced *Epo* or *Vegf* is inhibited by *Cited2* expression at the mRNA level in both Hep3B and HEK293 cells (Shin et al, 2008). In the present study, the mRNA level of *Cited2* was up-regulated in the liver, lung, and spleen of *E. baileyi*, and the expression of *Epo* did not show a significant difference in the kidney of laboratory rats, the liver of *E. baileyi*, and the kidney of *E. cansus*, suggesting that the expression of *Epo* in these tissues of *E. baileyi* and/or *E. cansus* is inhibited by *Cited2*, and insensitive to EPAS1. Therefore, the gain-of-function of EPAS1 in *E. baileyi* and *E. cansus* may contribute to their adaptation to the underground burrow environment by preventing erythrocytosis.

Fibrosis, characterized by the abnormal accumulation of ECM components, particularly collagens, is a defining feature of several chronic diseases, including idiopathic pulmonary fibrosis and liver cirrhosis. TGF-β1 and hypoxic conditions are well documented as key drivers of fibrosis, as they enhance the expression of ECM components and matrix-remodeling enzymes, such as procollagen prolyl and lysyl hydroxylases (An et al, 2024). The EPAS1 and TGF-β signaling pathways collaborate to drive glomerular fibrogenesis under normoxic conditions. Inhibition of HIF-1ɑ/2ɑ expression through siRNA reduced both basal and TGF-β1–induced type I collagen expression, and overexpression of a nondegradable form of HIF-ɑ enhanced collagen production, with EPAS1 demonstrating a more pronounced effect than HIF-1ɑ (Pei et al, 2019). Hypoxia and HIF not only affect the synthesis of collagen but also influence its cross-linking. Procollagen-lysine, 2-oxoglutarate 5-dioxygenase 2 encodes the sole lysyl hydroxylase isoform responsible for hydroxylating lysine residues in collagen telopeptides, a critical posttranslational modification necessary for the formation of stable cross-links. The expression of procollagen-lysine, 2-oxoglutarate 5-dioxygenase 2 is up-regulated by both hypoxia and TGF-β1. Studies indicate that intact HIF-binding sites are essential for TGF-β1 to exert its effects on suppressor of mother against decapentaplegic-binding sites (Lu et al, 2019). Consistent with previous reports, in HEK293T cells, transfection with the EPAS1-S480 plasmid significantly induced an increase in *Col1a1* and *Col3a1* mRNA levels compared with the EPAS1-T480. However, in primary LFs, TGF-β markedly up-regulated the expression levels of COL1A1 and F-actin in rats, whereas it had no significant effect on the fibroblasts of *E. Baileyi*. We speculate that the S480 variation enhances the stability of EPAS1 in plateau zokors, and may also improve the antifibrosis effects against TGF-β, hypoxia, and oxidative stress.

In the primary LFs of plateau zokor, the background expression level of *Cited2* mRNA was significantly higher than that of laboratory rats (Fig 5A). TGF-β treatment induced an increase in the *E. baileyi Cited2* mRNA level, while having no significant effect on laboratory rat cells (Fig 5C). The overexpression of *Cited2* inhibits the expression and activity of MMPs (such as MMP2, MMP3, and MMP9) (Lee et al, 2009), whereas some MMPs (such as MMP2, MMP3, and MMP8) are considered to play a clear profibrotic role in tissue fibrosis, such as pulmonary fibrosis (Pardo et al, 2016; Bormann et al, 2022; Chuliá-Peris et al, 2022). Therefore, we speculate that *Cited2* also plays an important role in antifibrosis in the primary LFs of plateau zokor, and the specific mechanisms underlying this phenomenon require further investigation.

The IFN pathway is primarily negatively regulated by hypoxia in tumor cells (Miar et al, 2020), but under certain conditions, hypoxia can also enhance this pathway, exerting a positive regulatory effect. For example, hypoxia reduces the pathogenicity of vesicular stomatitis virus by enhancing the IFN pathway, whereas inhibition of HIF increases cellular susceptibility to this viral infection (Arnaiz & Harris, 2022). Unexpectedly, loss-of-function variants of EPAS1 in the *S. galili* basalt population and gain-of-function variant of EPAS1 in *E. baileyi* both play negative roles in IFN signals. Low-dose transfection with the *S. galili* EPAS1 plasmid was used to mimic the loss-of-function variant of the EPAS1 in *S. galili* basalt population, and qRT-PCR analysis revealed that the mRNA levels of IFITs (interferon-induced proteins with tetratricopeptide repeats) such as Ifit1p1, Ifit3, and Ifit5 were significantly lower than those in the high-dose group (Fig 3G). IFITs play a key role in antiviral defense by binding and regulating both host and viral proteins and RNAs (Fensterl & Sen, 2015). IFIT3 silencing significantly improved cardiac function and reduced myocardial fibrosis and collagen content in mice with myocardial infarction (Sun et al, 2021). In *E. baileyi* LFs, mRNA expression in the IFN pathway was down-regulated under hypoxic conditions (Fig 5E). This pathway was suppressed at several levels of signaling, from transcription factors IRF1 and IRF3, INF receptors including Ifnar1, Ifnar2, and Ifngr2, to interferon-induced proteins including Ifit1, Ifitm2, and Ifitm3. Blockade of IFNAR1 reduces macrophage numbers compared with control mice and alleviates liver fibrosis, accompanied by increased hepatocyte proliferation and apoptosis (Park et al, 2025). In activated LX-2 cells (human hepatic stellate cells), IRF3 knockdown *via* siRNA significantly reduces the expression of type I collagen (Col1a1) and α-smooth muscle actin (α-SMA). Conversely, IRF3 overexpression up-regulates Col1a1 and α-SMA levels and further promotes hepatic stellate cell proliferation (Ni et al, 2016). The above information supports the concept that the negative regulation of the IFN pathway by the EPAS1 variants of *S. galili* basalt populations and *E. baileyi* may help reduce fibrosis and contribute to their adaptation to the hypoxic environment of subterranean caves.

**Figure S4.**
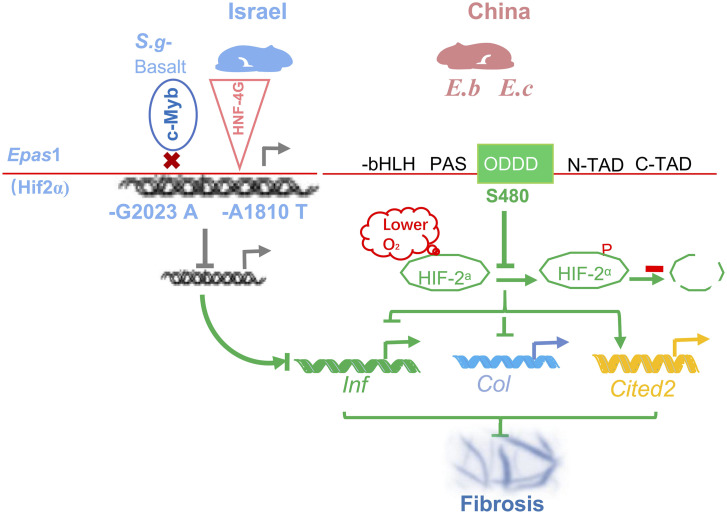
Schematic abstract for variants of EPAS1 contributes to hypoxia adaptation of the subterranean rodents.

In summary, we showed that the functional changes of *Epas1* in *E. baileyi*, *E. cansus*, *S. galili* chalk, and *S. galili* basalt have different evolutionary strategies in adaptation to hypoxia stress. Our study indicates an association between the *Epas1* variant and adaptation to subterranean habitats (Fig S4), and suggests that the study of subterranean mammalian species provides a powerful tool to understand the molecular mechanisms of hypoxic tolerance.

## Materials and Methods

### Animals

Animal protocols were approved by the IACUC of the School of Medicine, Zhejiang University (ZJU201304-1-01-025; ZJU20220102), and the University of Haifa, and followed the National Institutes of Health Guidelines. *E. baileyi* (250–350 g) were captured from a field near Haibei Research Station of the Alpine Meadow Ecosystem, Chinese Academy of Sciences (37°39′N, 101°19′E), in Qinghai, China. *E. cansus* (250–300 g, *E. cansus*) were captured from a field near Yan’an, North Shaanxi (36°36′N, 109°31′E), China. *R. pruinosus* (bamboo rat, 350-290 g) were obtained from Yangshuo (altitude 300 m, 34°53′N, 110°20′E), northeastern Guangxi, China. Adult male Sprague Dawley rats (150–200 g; Certification No. SCXK20190002) were purchased from the Experimental Animal Center, Zhejiang Academy of Medical Science. Blind mole rats, *S. galili* (2n = 52), were captured from a field in a defined region in the Upper Eastern Galilee Mountains. Ten individuals were collected from the Alma Pleistocene basalt plateau and 10 individuals from the Kerem Ben Zimra Senonian chalk (33°2.5′N, 35°29.2′E, 760 m above sea level). The animals were euthanized, and tissues were stored in liquid nitrogen immediately after capture. Genomic DNA and total RNA were extracted from the tissue. cDNA was obtained by reverse transcription.

### Animal treatment

Rats were placed in a hypobaric chamber (FLYDWC50-IIC; Avic Guizhou Fenglei Aviation Armament Co., Ltd) and exposed to normoxia (sea level, 21% O_2_, 100.08 kPa) or hypobaric hypoxia of 5,000-m (10.8% O_2,_ 54.02 kPa) and 7,000-m altitude (8.0% O_2_, 41.1 kPa) for 8 h (Hao et al, 2015).

### Cloning and point mutagenesis

Total RNA was extracted from the liver tissue of *S. galili*, *E. baileyi*, and *E. cansus* using RNAiso Plus (Takara) according to the manufacturer’s instructions. The first-strand cDNA was prepared from total RNA using an oligo-dT primer with PrimeScript reverse transcriptase (Takara). PCR primers were designed to amplify the regulatory region sequence and the cDNA fragment of *Epas1* (Table S3). PCR products were purified using an agarose gel DNA purification kit (Sangon Biotech) and ligated into a pGL3-basic vector. Site-directed mutagenesis of the expression vectors was performed using a FAST mutagenesis system kit (Transgene). All constructs were verified by sequencing.

Table S3. Primers for RT–PCR and qPCR.

### Cell culture and treatments

HEK293T, HeLa, and NCI-H1299 cells were grown in Dulbecco's Modified Eagle Medium or Roswell Park Memorial Institute 1640 Medium (Gibco), containing 10% (vol/vol) FBS (Gibco), 2 mM glutamine (Solarbio), 100 μg/ml streptomycin (Solarbio), and 100 U/ml penicillin (Solarbio) at 37°C in a humidified incubator with 5% (vol/vol) CO_2_. Cells were plated in a 12-well plate and transfected with vectors using LIPO3000 as described by the manufacturer’s instructions.

For the primary fibroblast culture, the fresh lung tissues of rats and *E. baileyi* were collected and stored in DMEM/F-12 medium. The primary fibroblasts were isolated with collagenase and cultured in the EMEM (Solarbio) containing 15% (vol/vol) FBS, 100 μg/ml streptomycin, and 100 U/ml penicillin at 37°C in a humidified incubator with 3% (vol/vol) O_2_ and 5% (vol/vol) CO_2_.

The hypoxia treatment was applied using the ProOx Model P110 and ProCO_2_ Model P120 hypoxia systems (BioSpherix). Cells were moved to the hypoxia incubator in which the O_2_ level was 0.1% for 24 h. Cells were treated with 10 μM Raf kinase inhibitor LY3009120, 10 μM ATM kinase inhibitor KU-55933, 20 μM PKA inhibitor H89 2HCL, 10 μg/ml specific inhibitor of AKT MK-2206 2HCL (Selleck), or 10 μM EPAS1 inhibitor belzutifan PT2977 (MCE) for 24 h, 5 ng/ml TGF-β (MCE) for 48 h, and 1 M H_2_O_2_ for 6 h before qRT-PCR, Western blot, and immunofluorescence analyses.

### qRT-PCR

Quantitative real-time polymerase chain reaction (qRT-PCR) was performed using SYBR Green PCR Master Mix (Toyobo) on ABI StepOne System (Applied Biosystems) with the primers listed in Table S3. A negative control was included in each run. Data were analyzed with the △△Ct (cycle threshold) method in which the amount of target RNA was adjusted to a reference (β-actin).

### Transient transfection

Transfection was performed using PolyJet reagent (SignaGen Laboratories). HEK293T, H1299, or HeLa cells were plated in 12-well plates at 180,000 cells per well. The cell density reaches the optimal 70–80% confluency at the time of transfection. The culture medium was replaced with a complete medium of serum and antibiotics for 30 min before transfection. Cells were cotransfected with a mixture of expression plasmids with promoter and luciferase reporter plasmids, or transfected with *E. baileyi* EPAS1 WT/mutant plasmids or *S. galili* EPAS1 high/low dose. Cells were harvested 48 h posttransfection.

### Transcriptome sequencing analysis

HEK293T cells were preseeded in 6-well plates in advance and then transfected with *E. baileyi* EPAS1 S480 and T480 plasmids, as the EPAS1 S480 group and EPAS1 T480 group. HEK293T cells were transfected with *S. galili* EPAS1 plasmids with high and low dose (3:1), as the EPAS1 high group and EPAS1 low group. The *E. baileyi* lung fibroblasts treated with hypoxia (0.1% O_2_, 24 h) were named the hypoxia group, and fibroblasts without any treatment served as the control.

Total RNA of all cells was extracted and purified using Total RNA Kit (Omega Bio-Tek) according to the manufacturer’s protocol. The total RNA amount was >1 mg, and RNA integrity was confirmed by electrophoresis with denaturing agarose gel. The transcriptomic analysis was conducted using Illumina NovaSeq 6000 (LC-Bio Technologies Co., Ltd.) following the standard procedures. The DEGs were selected by the mRNA expression level (|log2 fold change|>1, *P* <0.05) using the R package DESeq2 (http://www.bioconductor.org/packages/release/bioc/html/DESeq2.html), and then, GO/KEGG enrichment analyses of DEGs were conducted.

### Luciferase assay

A pGL3-promoter luciferase reporter construct driven by hypoxia response elements (HREs) was cotransfected into HEK293T cells with the pEGFP-EPAS1 (S480, S480A, S480T, S189, S189A, S189T, S189D) vector (Promega). pGL3-basic luciferase reporter constructs driven by the *S. galili Epas1* regulatory region were cotransfected into HEK293T cells with pRL-CMV (Promega), a non–hypoxia-responsive plasmid expressing *Renilla* luciferase. Luciferase activity was determined using Dual-Luciferase Reporter Assay System (Promega) in a Lumat3 luminometer (Titertek-Berthold), according to the manufacturer’s instructions.

### In vitro phosphorylation assay

Recombinant thioredoxin (Trx)-EPAS1 (H480S, H480A, and H480T) was incubated with nucleoprotein extracted from the mouse liver tissue in a total volume of 200 μl with phosphorylation buffer (25 mM Tris–HCl, pH 7.5, 10 mM MgCl_2_, 50 mM NaCl, and 50 μM ATP). Reactions were incubated at 30°C for 1 h and terminated by the addition of SDS sample buffer. Proteins were separated by 12% SDS–PAGE, and phosphorylation was visualized by phosphorylated Thr antibody (CST), phosphorylated Ser antibody (Boster), and thioredoxin antibody (Novagen).

### Flow cytometry

For apoptosis under stress, transfected cells were collected and stained with Annexin V–phycoerythrin (PE) and 7-aminoactinomycin D (7-AAD) (BD) for 15 min at room temperature. Then, the cells were subjected to flow cytometry (Beckman). Samples were gated to the staining of PE and 7-AAD. Apoptosis was calculated as the percentage of cells with positive PE staining. For cell-cycle arrest, cells were harvested and fixed in cold 70% ethanol at 4°C overnight. The next day, the DNA content was detected by staining cells with propidium iodide for 15 min at 37°C before being subjected to flow cytometry and analyzed by ModFit LT software.

### EMSA

The oligonucleotide probe (sense; Sangon) sequences used for EMSA were

-2023G 5′-TCA​AGG​GCT​TTG​GTA​GAA​TAC​AGT​TTT​AGG​TAC​CTT​G-3′,

-2023A 5′-TCA​AGG​GCT​TTG​GTA​GAA​TAC​AAT​TTT​AGG​TAC​CTT​G-3′,

-1810A 5′-TAA​AGT​ACA​AAT​AAT​AAA​AAA​AAT​TTT​GGT​CTT​CTG​AGA​CAG​GG-3′,

-1810T 5′-TAA​AGT​ACA​AAT​AAT​AAA​AAA​ATT​TTT​GGT​CTT​CTG​AGA​CAG​GG-3′.

Nonradioactive EMSAs were performed with nuclear proteins and biotin-labeled double-stranded probes, which were selected on the basis of predicted MYB- and HNF4G-binding sites in the regulatory region of the *Epas1* gene. EMSAs were performed using a LightShift Chemiluminescent EMSA kit (Thermo Fisher Scientific) according to the manufacturer’s instructions.

### Western blotting

Proteins were resolved by 12% SDS–PAGE and then electroblotted onto nitrocellulose membranes. Membranes were blocked for 1 h at room temperature in 5% nonfat dried milk/TBS/0.05% Tween-20 and probed with first antibodies (anti-HNF4G, 1:200, Proteintech; anti-MYB, 1:200, BioWorld; anti-GAPDH, 1:1,000, Boster; anti-EPAS1, 1:200, Boster; COL1A1, Zen-bio; β-actin, 1:1,000, Yeasen) overnight at 4°C. Membranes were then incubated with a corresponding secondary antibody (Boster) conjugated to horseradish peroxidase and incubated for 1 h at room temperature. The blots were developed using an ECL Plus kit from Beyotime.

### Immunofluorescence

The primary fibroblasts were cultured on slides in 12-well plates and treated with hypoxia (0.1% O_2_), TGF-β, or H_2_O_2_ as mentioned above. The cells were cleaned with PBS and fixed *via* 4% PFA. The slides were blocked for 1 h at room temperature in 5% goat serum and incubated in the primary antibody for COL1A1 (1:500, ab316222; Abcam) overnight at 4°C. The slides were incubated in a specific secondary antibody (Alexa Fluor 546, 1:1,000) for 2 h, and then incubated in 100 nM FITC-phalloidin (Solarbio) for 30 min, and the antifade mounting medium with DAPI (4',6-diamidino-2-phenylindole) for 10 min. The slides were imaged at 60× magnification using a confocal microscope (FV3000; Olympus): DAPI: excitation 405 nm, emission 420–460 nm; COL1A1: excitation 561 nm, emission 573–620 nm; F-actin: excitation 488 nm, emission 510–550 nm.

### Bioinformatics

The transcription factor–binding sites in upstream sequences of HIF-2α were identified by TFBIND (http://tfbind.hgc.jp/) and JASPAR (http://jaspar.genereg.net/) (Castro-Mondragon et al, 2022). To investigate whether the *Epas1* gene has undergone positive selection, we employed a branch-site model in the CodeML program (PAML V4.9) (Yang, 2007) to identify the positive selection sites along specific branches of the phylogenetic tree. Trees constructed using MEGA X (Kumar et al, 2018) with the NJ method were used as input for generating the CodeML site models of PAML [M1, M2, M8, and M7] to estimate the ratio of nonsynonymous (dN) to synonymous (dS) substitution (ω value). The significance of positive/diversifying selection is determined by comparing M1 (nearly neutral) versus M2 (positive selection) and M7 (beta distribution, ω > 1 disallowed) versus M8 (beta distribution, ω > 1 allowed) using the likelihood-ratio test. The Bayes–empirical Bayes method was used to detect potentially positively selected sites (Yang et al, 2005).

### Statistical analyses

The data are presented as the mean ± SEM. Different treatment groups were compared using a one-way ANOVA and paired *t* test. Fisher’s exact test was used for calculating differences in allelic distribution between the *Epas1* regulatory region of *S. galili* basalt and *S. galili* chalk. *P*-values <0.05 were considered significant.

## Supplementary Material

Reviewer comments

## Data Availability

All data needed to evaluate the conclusions in the article are present in the article and/or the Supplementary Materials. RNA-seq raw data for all the datasets have been submitted as raw sequence reads to NCBI and can be accessed via **BioProject** **ID** PRJNA1480854.

## References

[bib1] An X, Mao LY, Wang YJ, Xu QQ, Liu X, Zhang SZ, Qiao ZL, Li BW, Li F, Kuang ZR, (2024) Genomic structural variation is associated with hypoxia adaptation in high-altitude zokors. Nat Ecol Evol 8: 339–351. 10.1038/s41559-023-02275-7 38195998 PMC12301030

[bib2] Arnaiz E, Harris AL (2022) Role of hypoxia in the interferon response. Front Immunol 13: 821816. 10.3389/fimmu.2022.821816 35251003 PMC8895238

[bib3] Beall CM, Cavalleri GL, Deng L, Elston RC, Gao Y, Knight J, Li C, Li JC, Liang Y, McCormack M, (2010) Natural selection on* EPAS1 *(*HIF2alpha*) associated with low hemoglobin concentration in Tibetan highlanders. Proc Natl Acad Sci U S A 107: 11459–11464. 10.1073/pnas.1002443107 20534544 PMC2895075

[bib4] Befani C, Mylonis I, Gkotinakou IM, Georgoulias P, Hu CJ, Simos G, Liakos P (2013) Cobalt stimulates HIF-1-dependent but inhibits HIF-2-dependent gene expression in liver cancer cells. Int J Biochem Cell Biol 45: 2359–2368. 10.1016/j.biocel.2013.07.025 23958427 PMC3855297

[bib5] Bormann T, Maus R, Stolper J, Tarrés M, Brandenberger C, Wedekind D, Jonigk D, Welte T, Gauldie J, Kolb M, (2022) Role of matrix metalloprotease-2 and MMP-9 in experimental lung fibrosis in mice. Respir Res 23: 180. 10.1186/s12931-022-02105-7 35804363 PMC9270768

[bib6] Boyce WT, Sokolowski MB, Robinson GE (2020) Genes and environments, development and time. Proc Natl Acad Sci U S A 117: 23235–23241. 10.1073/pnas.2016710117 32967067 PMC7519332

[bib7] Cai ZY, Wang LY, Song XY, Tagore S, Li XF, Wang HH, Chen JR, Li KX, Frenkel Z, Gao DH, (2018) Adaptive transcriptome profiling of subterranean zokor, *Myospalax baileyi*, to high- altitude stresses in Tibet. Sci Rep 8: 4671. 10.1038/s41598-018-22483-7 29549310 PMC5856782

[bib8] Castro-Mondragon JA, Riudavets-Puig R, Rauluseviciute I, Lemma RB, Turchi L, Blanc-Mathieu R, Lucas J, Boddie P, Khan A, Pérez N, (2022) JASPAR 2022: The 9th release of the open-access database of transcription factor binding profiles. Nucleic Acids Res 50: D165–D173. 10.1093/nar/gkab1113 34850907 PMC8728201

[bib9] Chuliá-Peris L, Carreres-Rey C, Gabasa M, Alcaraz J, Carretero J, Pereda J (2022) Matrix metalloproteinases and their inhibitors in pulmonary fibrosis: EMMPRIN/CD147 comes into play. Int J Mol Sci 23: 6894. 10.3390/ijms23136894 35805895 PMC9267107

[bib10] Danial-Farran N, Nasser NJ, Beiles A, Brenner B, Sarig G, Nevo E (2017) Adaptive evolution of coagulation and blood properties in hypoxia tolerant *Spalax* in Israel. J Zool 303: 226–235. 10.1111/jzo.12480

[bib11] Darby IA, Hewitson TD (2016) Hypoxia in tissue repair and fibrosis. Cell Tissue Res 365: 553–562. 10.1007/s00441-016-2461-3 27423661

[bib12] Fensterl V, Sen GC (2015) Interferon-induced Ifit proteins: Their role in viral pathogenesis. J Virol 89: 2462–2468. 10.1128/JVI.02744-14 25428874 PMC4325746

[bib13] Fernandes MT, Calado SM, Mendes-Silva L, Braganca J (2020) CITED2 and the modulation of the hypoxic response in cancer. World J Clin Oncol 11: 260–274. 10.5306/wjco.v11.i5.260 32728529 PMC7360518

[bib14] Gale DP, Harten SK, Reid CD, Tuddenham EG, Maxwell PH (2008) Autosomal dominant erythrocytosis and pulmonary arterial hypertension associated with an activating HIF2 alpha mutation. Blood 112: 919–921. 10.1182/blood-2008-04-153718 18650473

[bib15] Gordan JD, Bertout JA, Hu CJ, Diehl JA, Simon MC (2007) HIF-2alpha promotes hypoxic cell proliferation by enhancing c-myc transcriptional activity. Cancer Cell 11: 335–347. 10.1016/j.ccr.2007.02.006 17418410 PMC3145406

[bib16] Gou X, Wang Z, Li N, Qiu F, Xu Z, Yan D, Yang S, Jia J, Kong X, Wei Z, (2014) Whole-genome sequencing of six dog breeds from continuous altitudes reveals adaptation to high-altitude hypoxia. Genome Res 24: 1308–1315. 10.1101/gr.171876.113 24721644 PMC4120084

[bib17] Guo YT, Zhang J, Xu DM, Tang LZ, Liu Z (2020) Phylogenomic relationships and molecular convergences to subterranean life in rodent family *Spalacidae*. Zool Res 42: 671–674. 10.24272/j.issn.2095-8137.2021.240 PMC8455469 34490760

[bib18] Hadid Y, Tzur S, Pavlicek T, Sumbera R, Skliba J, Lovy M, Fragman-Sapir O, Beiles A, Arieli R, Raz S, (2013) Possible incipient sympatric ecological speciation in blind mole rats (*Spalax*). Proc Natl Acad Sci U S A 110: 2587–2592. 10.1073/pnas.1222588110 23359700 PMC3574902

[bib19] Hao K, Kong FP, Gao YQ, Tang JW, Chen J, Evans AM, Lightman SL, Chen XQ, Du JZ (2015) Inactivation of corticotropin-releasing hormone-induced insulinotropic role by high-altitude hypoxia. Diabetes 64: 785–795. 10.2337/db14-0500 25277397

[bib20] Hu H, Petousi N, Glusman G, Yu Y, Bohlender R, Tashi T, Downie JM, Roach JC, Cole AM, Lorenzo FR, (2017) Evolutionary history of Tibetans inferred from whole-genome sequencing. PLoS Genet 13: e1006675. 10.1371/journal.pgen.1006675 28448578 PMC5407610

[bib21] Keith B, Johnson RS, Simon MC (2012) HIF1α and HIF2α: Sibling rivalry in hypoxic tumour growth and progression. Nat Rev Cancer 12: 9–22. 10.1038/nrc3183 PMC3401912 22169972

[bib22] Kietzmann T, Mennerich D, Dimova EY (2016) Hypoxia-inducible factors (HIFs) and phosphorylation: Impact on stability, localization, and transactivity. Front Cell Dev Biol 4: 11. 10.3389/fcell.2016.00011 26942179 PMC4763087

[bib71] Kumar S, Stecher G, Li M, Knyaz C, Tamura K (2018) MEGA X: Molecular evolutionary genetics analysis across computing platforms. Mol Biol Evol 35: 1547–1549. 29722887 10.1093/molbev/msy096 PMC5967553

[bib23] Lee JY, Taub PJ, Wang L, Clark A, Zhu LL, Maharam ER, Leong DJ, Ramcharan M, Li ZZ, Liu ZH, (2009) Identification of CITED2 as a negative regulator of fracture healing. Biochem Biophys Res Commun 387: 641–645. 10.1016/j.bbrc.2009.07.029 19607804 PMC3008352

[bib24] Lee P, Chandel NS, Simon MC (2020) Cellular adaptation to hypoxia through hypoxia inducible factors and beyond. Nat Rev Mol Cell Biol 21: 268–283. 10.1038/s41580-020-0227-y 32144406 PMC7222024

[bib25] Lei W, Rushton JJ, Davis LM, Liu F, Ness SA (2004) Positive and negative determinants of target gene specificity in myb transcription factors. J Biol Chem 279: 29519–29527. 10.1074/jbc.M403133200 15105423

[bib26] Li K, Hong W, Jiao H, Wang GD, Rodriguez KA, Buffenstein R, Zhao Y, Nevo E, Zhao H (2015) Sympatric speciation revealed by genome-wide divergence in the blind mole rat *Spalax*. Proc Natl Acad Sci U S A 112: 11905–11910. 10.1073/pnas.1514896112 26340990 PMC4586841

[bib27] Li K, Wang L, Knisbacher BA, Xu Q, Levanon EY, Wang H, Frenkel-Morgenstern M, Tagore S, Fang X, Bazak L, (2016) Transcriptome, genetic editing, and microRNA divergence substantiate sympatric speciation of blind mole rat, *Spalax*. Proc Natl Acad Sci U S A 113: 7584–7589. 10.1073/pnas.1607497113 27339131 PMC4941469

[bib28] Lin GH, Wang K, Deng XG, Nevo E, Zhao F, Su JP, Guo SC, Zhang TZ, Zhao H (2014) Transcriptome sequencing and phylogenomic resolution within *Spalacidae* (Rodentia). BMC Genomics 15: 32. 10.1186/1471-2164-15-32 24438217 PMC3898070

[bib29] Lou H, Lu Y, Lu D, Fu R, Wang X, Feng Q, Wu S, Yang Y, Li S, Kang L, (2015) A 3.4-kb copy-number deletion near EPAS1 is significantly enriched in high-altitude Tibetans but absent from the Denisovan sequence. Am J Hum Genet 97: 54–66. 10.1016/j.ajhg.2015.05.005 26073780 PMC4572470

[bib30] Lovy M, Skliba J, Hrouzkova E, Dvorakova V, Nevo E, Sumbera R (2015) Habitat and burrow system characteristics of the blind mole rat *Spalax galili* in an area of supposed sympatric speciation. PLoS One 10: e0133157. 10.1371/journal.pone.0133157 26192762 PMC4508111

[bib31] Lovy M, Skliba J, Sumbera R, Nevo E (2017) Soil preference in blind mole rats in an area of supposed sympatric speciation: Do they choose the fertile or the familiar? J Zool 303: 291–300. 10.1111/jzo.12489

[bib32] Lovy M, Sumbera R, Heth G, Nevo E (2020) Presumed ecological speciation in blind mole rats: Does soil type influence mate preferences? Ethol Ecol Evol 32: 1–14. 10.1080/03949370.2019.1646809

[bib33] Lu JL, Lam SM, Wan Q, Shi LX, Huo YN, Chen LL, Tang XL, Li BW, Wu XY, Peng K, (2019) High-coverage targeted lipidomics reveals novel serum lipid predictors and lipid pathway dysregulation antecedent to type 2 diabetes onset in normoglycemic Chinese adults. Diabetes Care 42: 2117–2126. 10.2337/dc19-0100 31455687

[bib34] Ma YF, Han XM, Huang CP, Zhong L, Adeola AC, Irwin DM, Xie HB, Zhang YP (2019) Population genomics analysis revealed origin and high-altitude adaptation of Tibetan pigs. Sci Rep 9: 11463. 10.1038/s41598-019-47711-6 31391504 PMC6685962

[bib35] Malik A, Korol A, Weber M, Hankeln T, Avivi A, Band M (2012) Transcriptome analysis of the spalax hypoxia survival response includes suppression of apoptosis and tight control of angiogenesis. BMC Genomics 13: 615. 10.1186/1471-2164-13-615 23148642 PMC3533650

[bib36] Maqueda M, Roca E, Brotons D, Soria JM, Perera A (2017) Affected pathways and transcriptional regulators in gene expression response to an ultra-marathon trail: Global and independent activity approaches. PLoS One 12: e0180322. 10.1371/journal.pone.0180322 29028836 PMC5640184

[bib37] Miar A, Arnaiz E, Bridges E, Beedie S, Cribbs AP, Downes DJ, Beagrie RA, Rehwinkel J, Harris AL (2020) Hypoxia induces transcriptional and translational downregulation of the type I IFN pathway in multiple cancer cell types. Cancer Res 80: 5245–5256. 10.1158/0008-5472.CAN-19-2306 33115807 PMC7611234

[bib38] Naas S, Schiffer M, Schödel J (2023) Hypoxia and renal fibrosis. Am J Physiol Cell Physiol 325: C999–C1016. 10.1152/ajpcell.00201.2023 37661918

[bib39] Nevo E (1961) Observations on Israeli populations of the mole rat, *Spalax ehrenbergi* (Nehring, 1898). Mammalia 25: 127–144.

[bib40] Nevo E, Ivanitskaya E, Filippucci MG, Beiles A (2000) Ecological speciation of *Spalax ehrenbergi* superspecies in Jordan: Four new putative species. Isr J Zool 142: 169. 10.1006/bijl.1999.0367

[bib41] Newman JH, Holt TN, Cogan JD, Womack B, Phillips JA, Li C, Kendall Z, Stenmark KR, Thomas MG, Brown RD, (2015) Increased prevalence of *EPAS1* variant in cattle with high-altitude pulmonary hypertension. Nat Commun 6: 6863. 10.1038/ncomms7863 25873470 PMC4399003

[bib42] Ni MM, Xu T, Wang YR, He YH, Zhou Q, Huang C, Meng XM, Li J (2016) Inhibition of IRF3 expression reduces TGF-β1-induced proliferation of hepatic stellate cells. J Physiol Biochem 72: 9–23. 10.1007/s13105-015-0452-6 26611114

[bib43] O'Brien KA, Simonson TS, Murray AJ (2020) Metabolic adaptation to high altitude. Curr Opin Endocr Metab Res 11: 33–41. 10.1016/j.coemr.2019.12.002

[bib44] Okumura F, Joo-Okumura A, Nakatsukasa K, Kamura T (2017) Hypoxia-inducible factor-2α stabilizes the von Hippel-Lindau (VHL) disease suppressor, Myb-related protein 2. PLoS One 12: e0175593. 10.1371/journal.pone.0175593 28394947 PMC5386292

[bib45] Pardo A, Cabrera S, Maldonado M, Selman M (2016) Role of matrix metalloproteinases in the pathogenesis of idiopathic pulmonary fibrosis. Respir Res 17: 23. 10.1186/s12931-016-0343-6 26944412 PMC4779202

[bib46] Park SJ, Garcia Diaz J, Comlekoglu T, Hahn YS (2025) Type I IFN receptor blockade alleviates liver fibrosis through macrophage-derived STAT3 signaling. Front Immunol 16: 1528382. 10.3389/fimmu.2025.1528382 40260261 PMC12009845

[bib47] Pei DQ, Shu XD, Gassama-Diagne A, Thiery JP (2019) Mesenchymal-epithelial transition in development and reprogramming. Nat Cell Biol 21: 44–53. 10.1038/s41556-018-0195-z 30602762

[bib48] Peng Y, Cui C, He Y, Ouzhuluobu, Zhang H, Yang D, Zhang Q, Bianbazhuoma, Yang L, He Y, (2017) Down-regulation of *EPAS1* transcription and genetic adaptation of Tibetans to high-altitude hypoxia. Mol Biol Evol 34: 818–830. 10.1093/molbev/msw280 28096303 PMC5400376

[bib49] Semenza GL (2011) Hypoxia. Cross talk between oxygen sensing and the cell cycle machinery. Am J Physiol Cell Physiol 301: C550–C552. 10.1152/ajpcell.00176.2011 21677261 PMC3174572

[bib50] Shah Z, Filonenko ES, Ramensky V, Fan C, Wang C, Ullah H, Zhang B, Volchkov P, Samokhvalov IM (2021) MYB bi-allelic targeting abrogates primitive clonogenic progenitors while the emergence of primitive blood cells is not affected. Haematologica 106: 2191–2202. 10.3324/haematol.2020.249193 32732364 PMC8327747

[bib51] Shams I, Avivi A, Nevo E (2005) Oxygen and carbon dioxide fluctuations in burrows of subterranean blind mole rats indicate tolerance to hypoxic-hypercapnic stresses. Comp Biochem Physiol A Mol Integr Physiol 142: 376–382. 10.1016/j.cbpa.2005.09.003 16223592

[bib52] Shao Y, Li JX, Ge RL, Zhong L, Irwin DM, Murphy RW, Zhang YP (2015) Genetic adaptations of the plateau zokor in high-elevation burrows. Sci Rep 5: 17262. 10.1038/srep17262 26602147 PMC4658562

[bib53] Shin DH, Li SH, Chun YS, Huang LE, Kim MS, Park JW (2008) CITED2 mediates the paradoxical responses of HIF-1alpha to proteasome inhibition. Oncogene 27: 1939–1944. 10.1038/sj.onc.1210826 17906695

[bib54] Skliba J, Lovy M, Koeppen SCW, Plestilova L, Vitamvas M, Nevo E, Sumbera R (2016) Activity of free-living subterranean blind mole rats *Spalax galili* (Rodentia: *Spalacidae*) in an area of supposed sympatric speciation. Biol J Linn Soc 118: 280–291. 10.1111/bij.12741

[bib55] Song S, Yao N, Yang M, Liu XX, Dong KZ, Zhao QJ, Pu YB, He XH, Guan WJ, Yang N, (2016) Exome sequencing reveals genetic differentiation due to high-altitude adaptation in the Tibetan cashmere goat (*Capra hircus*). BMC Genomics 17: 122. 10.1186/s12864-016-2449-0 26892324 PMC4758086

[bib56] Sun J, Zhang Q, Liu X, Shang X (2021) Downregulation of interferon-induced protein with tetratricopeptide repeats 3 relieves the inflammatory response and myocardial fibrosis of mice with myocardial infarction and improves their cardiac function. Exp Anim 70: 522–531. 10.1538/expanim.21-0060 34234081 PMC8614010

[bib57] Taylor CT, Scholz CC (2022) The effect of HIF on metabolism and immunity. Nat Rev Nephrol 18: 573–587. 10.1038/s41581-022-00587-8 35726016 PMC9208707

[bib58] Usui-Ouchi A, Aguilar E, Murinello S, Prins M, Gantner ML, Wright PE, Berlow RB, Friedlander M (2020) An allosteric peptide inhibitor of HIF-1α regulates hypoxia-induced retinal neovascularization. Proc Natl Acad Sci U S A 117: 28297–28306. 10.1073/pnas.2017234117 33106407 PMC7668029

[bib59] Wang XD, Angelis N, Thein SL (2018) MYB - A regulatory factor in hematopoiesis. Gene 665: 6–17. 10.1016/j.gene.2018.04.065 29704633 PMC10764194

[bib60] Wei DB, Wei L, Zhang JM, Yu HY (2006) Blood-gas properties of plateau zokor (*Myospalax baileyi*). Comp Biochem Physiol A Mol Integr Physiol 145: 372–375. 10.1016/j.cbpa.2006.07.011 16945563

[bib61] Xu SH, Li SL, Yang YJ, Tan JZ, Lou HY, Jin WF, Yang L, Pan XD, Wang JC, Shen YP, (2011) A genome-wide search for signals of high-altitude adaptation in Tibetans. Mol Biol Evol 28: 1003–1011. 10.1093/molbev/msq277 20961960

[bib62] Xu XH, Huang XW, Qun L, Li YN, Wang Y, Liu C, Ma Y, Liu QM, Sun K, Qian F, (2014) Two functional loci in the promoter of EPAS1 gene involved in high-altitude adaptation of Tibetans. Scientific Rep 4: 7465. 10.1038/srep07465 PMC4264014 25501874

[bib69] Yang Z (2007) PAML 4: Phylogenetic analysis by maximum likelihood. Mol Biol Evol 24: 1586–1591. 17483113 10.1093/molbev/msm088

[bib70] Yang Z, Wong WSW, Nielsen R (2005) Bayes empirical bayes inference of amino acid sites under positive selection. Mol Biol Evol 22: 1107–1118. 15689528 10.1093/molbev/msi097

[bib63] Yi X, Liang Y, Huerta-Sanchez E, Jin X, Cuo ZX, Pool JE, Xu X, Jiang H, Vinckenbosch N, Korneliussen TS, (2010) Sequencing of 50 human exomes reveals adaptation to high altitude. Science 329: 75–78. 10.1126/science.1190371 20595611 PMC3711608

[bib64] Zeng J, Wang Z, Shi Z (1984) Metabolic characteristics and some physiological parameters of mole rat (*Myospalax baileyi*) in alpine area. Acta Biol Pla Sin 3: 163–171.

[bib65] Zhang W, Fan Z, Han E, Hou R, Zhang L, Galaverni M, Huang J, Liu H, Silva P, Li P, (2014) Hypoxia adaptations in the grey wolf (*Canis lupus chanco*) from Qinghai-Tibet Plateau. PLoS Genet 10: e1004466. 10.1371/journal.pgen.1004466 25078401 PMC4117439

[bib66] Zhao Y, Ren JL, Wang MY, Zhang ST, Liu Y, Li M, Cao YB, Zu HY, Chen XC, Wu CI, (2013) Codon 104 variation of p53 gene provides adaptive apoptotic responses to extreme environments in mammals of the Tibet plateau. Proc Natl Acad Sci U S A 110: 20639–20644. 10.1073/pnas.1320369110 24297887 PMC3870672

[bib67] Zhao Y, Tang JW, Yang Z, Cao YB, Ren JL, Ben-Abu Y, Li K, Chen XQ, Du JZ, Nevo E (2016) Adaptive methylation regulation of p53 pathway in sympatric speciation of blind mole rats, *Spalax*. Proc Natl Acad Sci U S A 113: 2146–2151. 10.1073/pnas.1522658112 26858405 PMC4776458

[bib68] Zhou W, Dou F (1990) Studies on activity and home range of plateau zokor. Acta Theriol Sinica 10: 31–39.

