# Supplementary material for: Variants of Epas1 contribute to hypoxia adaptation in the subterranean rodents Eospalax and Spalax
Source: Life Sci Alliance. 2026 Jul 20;9(9):e202603622. doi: 10.26508/lsa.202603622 (PMC13386345; doi:10.26508/lsa.202603622)
Supplement: Supplementary file 1 [file LSA-2026-03622_TableS1.docx]

**Table S1.**

Likelihood ratio test (LRT) of positive selection in the *Epas1* gene for vertebrates.

| **Model** | **np** | **lnL** | **κ** | **ω0** | **ω1** | **ω2** | **null** | **LRT** | **Positively selected sites (BEB)** |
| --- | --- | --- | --- | --- | --- | --- | --- | --- | --- |
| M1 | 92 | -38865.13 | 2.70 | 0.097  (73.64%) | 1.000  (26.36%) |  |  |  | None |
| M2 | 94 | -38865.13 | 2.70 | 0.097  (73.64%) | 1.000  (26.36%) |  | M1 | <0.01 | 27A (p=0.979)* |
| M3 | 95 | -38486.64 | 2.48 | 0.011  (37.26%) | 0.162  (37.36%) | 0.594  (25.38%) |  |  | None |
| M7 | 92 | -38470.87 | 2.48 | p =0.394, q =1.362 | | |  |  |  |
| M8 | 94 | -38445.45 | 2.51 | p0 =0.971, p =0.410, q =1.567,  (p1 =0.02892), ω=60.520 | | | M7 | 50.84** | 27A (p=0.997)** |

*p-value<0.05, **p-value<0.01
