# Supplementary material for: Variants of Epas1 contribute to hypoxia adaptation in the subterranean rodents Eospalax and Spalax
Source: Life Sci Alliance. 2026 Jul 20;9(9):e202603622. doi: 10.26508/lsa.202603622 (PMC13386345; doi:10.26508/lsa.202603622)
Supplement: Supplementary file 2 [file LSA-2026-03622_TableS2.docx]

**Table S2.**

Branch-site test of positive selection (branch-site model A, test 2) in the *Epas1* gene of *Eospalax*, *Spalax*, and naked carp.

| **Partition** | **np** | **LRT** | **ω parameters** | | **Positively selected sites (BEB)** |
| --- | --- | --- | --- | --- | --- |
|  |  |  | foreground | background |  |
| *E. baileyi* |  | <0.01 | ω0= 0.097(73.64%)  ω1= 1.000(26.36%)  ω2a= 1.000 (0%)  ω2b= 1.000 (0%) | ω0=0.096(73.64%)  ω1=1.000(26.36%)  ω2a=0.097(0%)  ω2b=1.000(0%) |  |
| *E. cansus* | 94 | <0.01 | ω0= 0.096(73.39%)  ω1= 1.000(26.36%)  ω2a= 24.41(0.21%)  ω2b= 24.416(0.08%) | ω0=0.096(73.39%)  ω1=1.000(26.33%)  ω2a=0.096(0.21%)  ω2b=1.000(0.08%) | 628A (p=0.895) |
| *Spalax* | 94 | <0.01 | ω0= 0.097(73.64%)  ω1= 1.000(26.36%)  ω2a= 1.000 (0%)  ω2b= 1.000 (0%) | ω0= 0.096(73.64%)  ω1= 1.000(26.36%)  ω2a= 0.096 (0%)  ω2b= 1.000 (0%) | None |
| Naked carp | 94 | <0.01 | ω0= 0.093(44.00%)  ω1= 1.000(15.94%)  ω2a= 1.000 (29.41%)  ω2b= 1.000 (10.65%) | ω0= 0.093(44.00%)  ω1= 1.000(15.94%)  ω2a= 0.093 (29.41%)  ω2b= 1.000 (10.65%) | 225W  (p=0.989) * |

*p-value<0.05
