# Supplementary material for: Variants of Epas1 contribute to hypoxia adaptation in the subterranean rodents Eospalax and Spalax
Source: Life Sci Alliance. 2026 Jul 20;9(9):e202603622. doi: 10.26508/lsa.202603622 (PMC13386345; doi:10.26508/lsa.202603622)
Supplement: Supplementary file 3 [file LSA-2026-03622_TableS3.docx]

**Table S3.**

Primers for RT-PCR and q-PCR

| **Primer for** | **Sequence** | **Amplified fragments** |
| --- | --- | --- |
| Cloning | CTCGAGCTGGTATCTGGCTCACACT | *Epas1* promoter sequence |
|  | AAGCTTAGGTGGAAGTTT GCGGACGG |  |
|  | CCGCTCGAGCTTTGTGCTATCTGGTTCT |  |
|  | CCCAAGCTTCCTT TTCAACCCCACGG |  |
|  | CCAGCGCCTAAGGAACCCAG | *Epas1* cDNA fragment |
|  | AGCAGGTAAA ACAACTATGGAT |  |
|  | GGAATTCATGATGGCGACAGCTGACAAGGAGAAG |  |
|  | GAAGCTTGCCACCATGACAGCTGACAAGGAGAAG |  |
| qPCR | TGGCTCAGTTCAGCAGGAAC | *S. galili* *Epas1* |
|  | GCAGGGTCCGAGGTATTC |  |
|  | GGTGACCCAAGATGGTGATA | *E. baileyi*, *E. cansus,* and rat *Epas1* |
|  | GTCGCAAGGATGAGTGAAGT |  |
|  | GATTGGACCGCATCAAGGAG | *Cited2* |
|  | GGGGAGGGTGATTTCTTTCG |  |
|  | GGCTTTGGCGTTTCAGGG | *S. galili* *Hif-1α* |
|  | GCAGCGATGACACCGAAAC |  |
|  | ACCATCACTGTCACTGCCAC | rat *Hif-1α* |
|  | TCCTGCTCTGTCTGGTGAGG |  |
|  | TGCCAAAAGAGGTGGCTATGT | *E. baileyi* and *E. cansus* *Hif-1α* |
|  | AATGCACTGTGGTTGAGAGTT |  |
|  | GAACCGAAGCACCGAAACC | *S. galili* and rat *Glut1* |
|  | GAGAAGCCAAGCGAAGCAAT |  |
|  | GACCCTGCATCTCATTGGTCT | *E. baileyi* and *E. cansus* *Glut1* |
|  | AACAGCTCGGCCACAATGAA |  |
|  | GGAAGGGTGTTAGACTCTGGAA | *S. galili* *Vegf* |
|  | TGAGAACCTGGGCTGTGAAAT |  |
|  | ACGAAAGCGCAAGAAATCCC | rat *Vegf* |
|  | GCAACGCGAGTCTGTGTTTT |  |
|  | TGAGCTTCCTACAGCACAACA | *E. baileyi* and *E. cansus* *Vegf* |
|  | GGCTCACAGTGAACACTCCA |  |
|  | CCGGCCAGTGAGTGTAAAAA | *E. baileyi*, *E. cansus,* and rat Vegfr-2 |
|  | CTCCCTGCTTTTACTGGGCAT |  |
|  | GCTCTGCTATGTGGCTCT | β-actin |
|  | CGCAAGACTCCATACCC |  |
|  | TGTCGCCTACTTGGATGTGC  CCTTGCAGGCCAGTAGTGTT  CTAAAGCCCAGCATCTC  TTCATAGCAGCAACCAG  AACCCAGAGGAAGTGGCAAT  GACAGTGAAGGCTCAAAGATGG  GATGGGCACTGTAAAGGAGA  GCAGGTCCACATCATTCG  GCCTCTCGCCCATATGTTTG  AAGCAGCCTGATGTCTCCTT | Rat *Crot*  *E. baileyi*/*E. cansus*/*S. galili* *Crot*  *Cd36*  *Cpt1*  *Acsl1* |
