# Supplementary material for: Variants of Epas1 contribute to hypoxia adaptation in the subterranean rodents Eospalax and Spalax
Source: Life Sci Alliance. 2026 Jul 20;9(9):e202603622. doi: 10.26508/lsa.202603622 (PMC13386345; doi:10.26508/lsa.202603622)
Supplement: Reviewer comments [file LSA-2026-03622_review_history.pdf]

Variants of EPAS1 Contribute to Hypoxia Adaptation in the Subterranean Rodents Eospalax and Spalax

Yibin Cao, Mengchen Zhang, Xin Li, Tianfu Yu, Yi Li, Hua Wang, Mengqi Liu, Xiaokang Xu, Fangyuan Xia, Kexin Li, Qiang Chen, Honghao Yu, Dengbang Wei, Yang Zhao, Eviatar Nevo, Ji-Zeng Du, and Xue-Qun Chen
DOI: https://doi.org/10.26508/lsa.202603622

Corresponding author(s): Xue-Qun Chen, Zhejiang University and Yibin Cao, Zhejiang Normal University

Review Timeline:

Submission Date:	2026-01-08
Editorial Decision:	2026-03-19
Revision Received:	2026-05-03
Editorial Decision:	2026-06-11
Revision Received:	2026-06-19
Accepted:	2026-06-26

Scientific Editor: Sarita Hebbar

Transaction Report:

(Note: With the exception of the correction of typographical or spelling errors that could be a source of ambiguity, letters and reports are not edited. The original formatting of letters and referee reports may not be reflected in this compilation.)

March 19, 2026

Re: Life Science Alliance manuscript #LSA-2026-03622-T

Xue-Qun Chen
Zhejiang University
Neurobiology and Physiology
School of medicine, Research building C-507
Hangzhou, Zhejiang
China

Dear Dr. Chen,

Thank you for submitting your manuscript entitled "Variants of EPAS1 contribute to hypoxia adaptation of the subterranean rodents Myospalax and Spalax" to Life Science Alliance.

Your manuscript was evaluated by two expert reviewers whose comments are appended below. As you will read, both reviewers have commented that your study is of potential value. However they have also raised several concerns that preclude publication of the work in its current form.

Based on their overall assessment, we invite you submit a revised version addressing both reviewers' concerns related to
- using consistent nomenclature
- extending the methods and description of the results and
- reorganising the discussion to better explain your findings in these model species, followed by extracting relevant findings from these comparisons, and clearly stating limitations wherever appropriate.

I would be happy to discuss the revision in more detail via email or phone/videoconferencing. Please let me know which option you prefer, if any.

To upload the revised version of your manuscript, please log in to your account: https://lsa.msubmit.net/cgi-bin/main.plex

You will be guided to complete the submission of your revised manuscript and to fill in all necessary information. Please get in touch in case you do not know or remember your login name.

The typical timeframe for revisions is three months. Please note that papers are generally considered through only one revision cycle, so strong support from the referees on the revised version is needed for acceptance.

While you are revising your manuscript, please also attend to the below editorial points to help expedite the publication of your manuscript. Please direct any editorial questions to the journal office. When submitting the revision, please include a letter addressing the reviewers' comments point by point.

Thank you for this interesting contribution to Life Science Alliance. We hope that the comments below will prove constructive as your work progresses, and we are looking forward to receiving your revised manuscript.

Sincerely,

Sarita Hebbar, PhD
Scientific Editor
Life Science Alliance
http://www.lsajournal.org

A. THESE ITEMS ARE REQUIRED FOR REVISIONS

-- A letter addressing the reviewers' comments point by point.

-- An editable version of the final text (.DOC or .DOCX) is needed for copyediting (no PDFs).

-- High-resolution figure, supplementary figure and video files uploaded as individual files: See our detailed guidelines for preparing your production-ready images, https://www.life-science-alliance.org/authors

-- Summary blurb (enter in submission system): A short text summarizing in a single sentence the study (max. 200 characters including spaces). This text is used in conjunction with the titles of papers, hence should be informative and complementary to the title and running title. It should describe the context and significance of the findings for a general readership; it should be written in the present tense and refer to the work in the third person. Author names should not be mentioned.

-- By submitting a revision, you attest that you are aware of our payment policies found here: https://www.life-science-alliance.org/copyright-license-fee

B. MANUSCRIPT ORGANIZATION AND FORMATTING:

Full guidelines are available on our Instructions for Authors page, https://www.life-science-alliance.org/authors

We encourage our authors to provide original source data, particularly uncropped/-processed electrophoretic blots and spreadsheets for the main figures of the manuscript. If you would like to add source data, we would welcome one PDF/Excel-file per figure for this information. These files will be linked as supplementary "Source Data" files.

IMPORTANT: It is Life Science Alliance policy that if requested, original data images must be made available. Failure to provide original images upon request will result in unavoidable delays in publication. Please ensure that you have access to all original microscopy and blot data images before submitting your revision.

Reviewer #1 (Comments to the Authors (Required)):

In this manuscript, Cao and co-workers compare the Epas1 gene, which encodes the HIF-2a protein, across Myospalax rodents from China and Spalax rodents from Israel. These rodents regularly experience hypoxia in their natural habitats. Therefore, the goal of the study was to identify functional changes that contribute to hypoxic tolerance. The authors report a number of changes in regulatory and coding regions of the gene, which, however, differ across species. They conclude that there are multiple paths to the evolution of hypoxic tolerance among the species studied.

This is a generally well-written and easy-to-understand manuscript. I found that the conclusions of this study were supported by the results. My main criticism is that computational analyses are described in too little detail for readers to be able to understand them and even reproduce them. Some examples are in lines 137-141 ("In the M2 and M8 model of the branch test..."), 171-172 ("Bioinformatic analysis predicted that..."), and in the caption of Fig. 1 ("Phylogenetic tree for HIF2-a constructed..."). I strongly recommend that the authors explicitly report the tools they used, their version numbers, the parameters of the models that they applied, along with explanations as to why their choices are reasonable for their data.

Other comments:

- It is no fault of the authors, but the different names for the gene and the protein can be confusing to readers. I would reiterate at the beginning of the Discussion section that HIF-2a refers to the protein encoded by the Epas1 gene. Also, in line 123, shouldn't "regulatory regions of HIF-2a" be "regulatory regions of Epas1"?

- In line 93, the authors refer to SNPs in the intron of Epas1 that are associated with adaptation to hypoxia in Tibetans. There is no discussion of how many introns this gene has, nor which intron these SNPs have been detected in.

- In general, I would strongly advise the authors not to use red and green in the same figure panel, as they are perceived identically by certain colorblind readers. There are several colorblind-friendly color palettes online that the authors can use.

- I find Figure 1E extremely detailed, including country names and country borders that extend all the way to the South China Sea. To me, all these pieces of information are quite distracting from the goal of the figure, which is to show where the species of focus are found. I would strongly recommend that the authors replace this panel with something like a topographic map that does not include country borders. I believe that the average reader knows where China and Israel lie and, in any case, borders that include sea regions are not relevant to a study of terrestrial species. Also, there is a typo in the Figure ("Isreal").

- There are a couple of occurrences of "!!! INVALID CITATION !!!".

- At the end of the Discussion, the authors mention that the species they studied had very different changes in their Epas1 gene. A main text figure that summarises these changes separately for each species (in different panels) would be very helpful. Currently, the authors have a figure like this in the Supplementary Materials (Figure S4), but I find it very hard to tell which changes correspond to which species just by looking at this figure.

Reviewer #2 (Comments to the Authors (Required)):

Subterranean rodents are biologically interesting since they have successfully adapted to withstand abiotic stressors like hypoxia, hypercapnia and darkness in their underground habitats. Understanding the underlying genetic and physiological mechanisms of these adaptations may prospectively be important for developing novel biomedical strategies to combat stress-associated diseases in humans. The general topic of the present manuscript by Cao et al. is therefore potentially of broader scientific interest. The authors have studied molecular changes in the sequence and expression of EPAS1, a known master regulator of hypoxia response, in geographically separate mole rat species, the Israeli blind mole rat Spalax (with two soil-adapted forms undergoing incipient speciation) and high-altitude rodents of the genus Myospalax, living in China.

The following aspects appear as the key novel findings of this paper:

• In Israel, two genetic forms of the blind mole rat Spalax galili, which are adapted to either chalk or basalt, are differentiated by nucleotide substitutions in the candidate upstream regulatory elements of EPAS1. These variants alter the binding of specific transcription factors (c-Myb, HNF4G), which may be causal to expression level changes of EPAS1 in Spalax tissue.

• The Plateau zokor (Myospalax) and related taxa feature a specific EPAS1 variant (T480S) which is neither present in the hypoxia-adapted Spalax and nor in other hypoxia-sensitive rodents. This amino acid replacement eliminates a key phosphorylation site within the oxygen-dependent degradation domain, which increases protein stability and alters the hypoxia response.

• It is hypothesized that divergent evolutionary changes in the EPAS1-mediated hypoxia response of the two rodent lineages (i.e. mutations in EPAS1 regulatory elements in Spalax and mutations in therEPAS1 coding region in zokors) converge in their function to downregulate important immune-modulating pathways and fibrotic processes, thus contributing to hypoxia tolerance.

While there are such novel data in this manuscript that are of potential interest (mainly for researchers working on hypoxia and these model species), the paper shows many shortcomings in the way the data are presented here. In particular, the discussion currently reads somewhat chaotic and should be strictly arranged (e.g. by first discussing the two experimental systems separately, then making a synthesis) and also shortened to highlight the most important aspects.

I cannot recommend the manuscript for publication unless major revision takes place.

The following individual points should be considered:

• line 114: how were the regulatory sequences identified? What makes them bona fide promotors?

• Fig 1A: at the promotor variant sites, the allele frequency differences between the basalt and chalk populations of Sga are seemingly based on very few individuals (n=10). Do the observed differences excess genome-wide divergence? How can you make sure the variants are population-diagnostic? Also state the limitation that there is currently no evidence linking the regulatory sequence variants to reproductive isolation, speciation or fitness differences between populations.

• The effects of EPAS1 gene promotor variants have been studied in heterologous cells (HEK293T). Is there possibility to study transcription factor binding via ChiP-Seq in native Spalax tissue? If not, a sentence describing this limitation should be added.

• Fig 2A, qRT-PCR: why was actin chosen as reference gene? Is stable expression of actin demonstrated? Lack of variants within primer binding sites verified (this could heavily influence quantification)? How many animals/biological replicates were used? (give number in legend)

• lines 178-184: the experiment is not explained understandably. RNA-Seq ! was performed on exactly what? What are "low- and high dose" plasmids??

• RNA-Seq data should be uploaded to public database and acc. nos. given in the paper.

• Stability of mutated EPAS1: there are no direct measurements of protein half-life or degradation kinetics. This limitation should be mentioned.

• The causality between EPAS1 action and interferon signaling was inferred from a remote cell culture model. Is it possible to perform RNA-Seq on tissue from basalt and chalk specimen to measure interferon pathway gene activity in primary cells?

• Fig 5: why are mRNA and protein expression levels for the different collagen variants not measured side-by-side? (Col3a1 mRNA, Col1A1 protein) Is it safe that the anti-col1A1 antibodies robustly bind to the zokor protein?

• TGFbeta pathway involvement in zokor was not directly measured. This should be mentioned as limitation.

- Evidence for an antifibrotic condition in zokor versus rat is limited: can you identify matching RNA-Seq data from rat to demonstrate species-specific and genome-wide response of fibrosis-associated genes?

Additional points for revision:

- The text is not very smoothly written, also containing jumps of thoughts, making it cumbersome to read. Language editing is advised. In Results, each new section should shortly introduce the following line of experiments and why they were made...

- Avoid jumping between gene designations EPAS1 and Hif2a.

- Reference section: many citations are incomplete and require thorough editing. Inside the text there are wrong citation names, e.g. Katie O'Brien 2020 should be O'Brien and Murray.

- For species abbreviations in text and figures, please stick to accepted rules (first letter of genus plus initial 2 letters of species designation. E. g. use Sga for Spalax galili). Avoid unnecessary and confusing punctuation like in "S.g." or "M.B.".

- line 39: "link transcriptional activity and the mRNA transcription" > ????

- line 68: delete "dramatic"

- line 76: higher than Mc, I guess?

- line 80: use latin symbol for alpha throughout

- line 89: any newer review available?

- line 106: instead of "meanwhile" you may use "In parallel lines of investigation"

- lines 107-110 are not clearly phrased

- line 132-133: why are bamboo rats introduced here? Define "R.p."

- line 137 lacks introduction to the performed positive selection test applications. There is also nothing to be found in the MM section.

- line 167: "variant of EPAS1 regulates promotor activity" >>>> really??

- line 167/168: I do not think the DLRA measured transcription of EPAS1

- lines 174-175: what was done exactly? Description is unclear....

- line 261: you certainly mean "mutation hot spot"

- lines 262-263: the sentence is messed up: variants in the regulatory region...regulate the expression of target genes....via multiple transcription factors (????)

Response for LSA-2026-03622-T:

May 1, 2026
Sarita Hebbar, PhD. Scientific Editor
Life Science Alliance; http://www.lsajournal.org

Dear Dr. Sarita Hebbar,

Many thanks for your hard work and the reviewers' comments. We have addressed them one by one in blue as following.

Best Regards,
XQ Chen

Reviewer #1 (Comments to the Authors (Required)):
In this manuscript, Cao and co-workers compare the Epas1 gene, which encodes the HIF-2a protein, across Myospalax rodents from China and Spalax rodents from Israel. These rodents regularly experience hypoxia in their natural habitats. Therefore, the goal of the study was to identify functional changes that contribute to hypoxic tolerance. The authors report a number of changes in regulatory and coding regions of the gene, which, however, differ across species. They conclude that there are multiple paths to the evolution of hypoxic tolerance among the species studied.

Main criticism:

This is a generally well-written and easy-to-understand manuscript. I found that the conclusions of this study were supported by the results. My main criticism is that computational analyses are described in too little detail for readers to be able to understand them and even reproduce them. Some examples are in lines 137-141 ("In the M2 and M8 model of the branch test..."), 171-172 ("Bioinformatic analysis predicted that..."), and in the caption of Fig. 1 ("Phylogenetic tree for HIF2-a constructed..."). I strongly recommend that the authors explicitly report the tools they used, their version numbers, the parameters of the models that they applied, along with explanations as to why their choices are reasonable for their data.

We agree with these critical review comments. We have added detailed information for computational analyses of transcription factor binding sites, Phylogenetic tree and positive selection analysis in the **Methods** section.

"Bioinformatics:

The transcription factor binding sites in upstream sequences of HIF-2α were identified by TFBIND (http://tfbind.hgc.jp/) and JASPAR (http://jaspar.genereg.net/) (Castro-Mondragon et al, 2022). To investigate whether the *Epas*1 gene has undergone positive selection, we employed branch-site model in the CodeML program (PAML V4.9) (Yang, 2007) to identify the positive selection sites along specific branches of the phylogenetic tree. Trees constructed using MEGA X (Kumar et al, 2018) with NJ and ML method were used as input for generating the CodeML site models of PAML [M1, M2, M8, and M7] to estimate the ratio of non-synonymous (dN) to synonymous (dS) substitution (ω value). The significance of positive/diversifying selection is determined by comparing M1 (nearly neutral) vs M2 (positive selection) and M7 (beta distribution, ω > 1 disallowed) vs M8 (beta distribution, ω > 1 allowed)

using the likelihood ratio test (LRT). The Bayes empirical Bayes method was used to detect potentially positively selected sites (Yang et al, 2005) ".

Kumar S, Stecher G, Li M, Knyaz C, Tamura K (2018) MEGA X: Molecular Evolutionary Genetics Analysis across Computing Platforms. *Mol Biol Evol* **35:** 1547-1549
Yang Z (2007) PAML 4: Phylogenetic analysis by maximum likelihood. *Mol Biol Evol* **24:** 1586-1591
Yang Z, Wong WSW, Nielsen R (2005) Bayes empirical Bayes inference of amino acid sites under positive selection. *Mol Biol Evol* **22:** 1107–1118

Other comments:

(1) It is no fault of the authors, but the different names for the gene and the protein can be confusing to readers. I would reiterate at the beginning of the Discussion section that HIF-2a refers to the protein encoded by the Epas1 gene. Also, in line 123, shouldn't "regulatory regions of HIF-2a" be "regulatory regions of Epas1"?

We agreed this point. We have reiterated at the beginning of the Discussion section that HIF-2a refers to the protein encoded by the *Epas*1 gene in MS, and revised the protein and gene name respectively.

(2) In line 93, the authors refer to SNPs in the intron of Epas1 that are associated with adaptation to hypoxia in Tibetans. There is no discussion of how many introns this gene has, nor which intron these SNPs have been detected in.

There are 15 introns in Tibetans and *S.galili Epas*1, and one SNP in intron 1 and another SNP in intron 2 of Tibetans *Epas*1 were identified as significant SNPs that correlated with a lower-than-expected Hb concentration (Hanaoka et al, 2012).

Hanaoka M, Droma Y, Basnyat B, Ito M, Kobayashi N, Katsuyama Y, Kubo K, Ota M (2012) Genetic variants in EPAS1 contribute to adaptation to high-altitude hypoxia in Sherpas. *PLoS One* **7:** e50566

(3) In general, I would strongly advise the authors not to use red and green in the same figure panel, as they are perceived identically by certain colorblind readers. There are several colorblind-friendly color palettes online that the authors can use.

Thank you for pointing this out. We changed the color in the figures (Fig.1-Fig 5.) to make them colorblind-friendly.

(4) I find Figure 1E extremely detailed, including country names and country borders that extend all the way to the South China Sea. To me, all these pieces of information are quite distracting from the goal of the figure, which is to show where the species of focus are found. I would strongly recommend that the authors replace this panel with something like a topographic map that does not include country borders. I believe that the average reader knows where China and Israel lie and, in any case, borders that include sea regions are not relevant to a study of terrestrial species. Also, there is a typo in the Figure ("Isreal").

We replaced the map with a less detailed map. We also corrected the typo.

(5) There are a couple of occurrences of "!!! INVALID CITATION !!!".

Thank you for catching these. We re-inserted the references.

(6) At the end of the Discussion, the authors mention that the species they studied had very different changes in their Epas1 gene. A main text figure that summarises these changes separately for each species (in different panels) would be very helpful. Currently, the authors have a figure like this in the Supplementary Materials (Figure S4), but I find it very hard to tell which changes correspond to which species just by looking at this figure.

We have revised and summarized the information indicating the species corresponding to each change (Figure S4), now it looks clear.

Reviewer #2 (Comments to the Authors (Required)):
Subterranean rodents are biologically interesting since they have successfully adapted to withstand abiotic stressors like hypoxia, hypercapnia and darkness in their underground habitats. Understanding the underlying genetic and physiological mechanisms of these adaptations may prospectively be important for developing novel biomedical strategies to combat stress-associated diseases in humans. The general topic of the present manuscript by Cao et al. is therefore potentially of broader scientific interest. The authors have studied molecular changes in the sequence and expression of EPAS1, a known master regulator of hypoxia response, in geographically separate mole rat species, the Israeli blind mole rat Spalax (with two soil-adapted forms undergoing incipient speciation) and high-altitude rodents of the genus Myospalax, living in China.
The following aspects appear as the key novel findings of this paper:
• In Israel, two genetic forms of the blind mole rat Spalax galili, which are adapted to either chalk or basalt, are differentiated by nucleotide substitutions in the candidate upstream regulatory elements of EPAS1. These variants alter the binding of specific transcription factors (c-Myb, HNF4G), which may be causal to expression level changes of EPAS1 in Spalax tissue.
• The Plateau zokor (Myospalax) and related taxa feature a specific EPAS1 variant (T480S) which is neither present in the hypoxia-adapted Spalax and nor in other hypoxia-sensitive rodents. This amino acid replacement eliminates a key phosphorylation site within the oxygen-dependent degradation domain, which increases protein stability and alters the hypoxia response.
• It is hypothesized that divergent evolutionary changes in the EPAS1-mediated hypoxia response of the two rodent lineages (i.e. mutations in EPAS1 regulatory elements in Spalax and mutations in therEPAS1 coding region in zokors) converge in their function to downregulate important immune-modulating pathways and fibrotic processes, thus contributing to hypoxia tolerance.
While there are such novel data in this manuscript that are of potential interest (mainly for researchers working on hypoxia and these model species), the paper shows many shortcomings in the way the data are presented here.

Main Concerns:
In particular, the discussion currently reads somewhat chaotic and should be strictly arranged (e.g. by first discussing the two experimental systems separately, then making a synthesis) and also shortened to highlight the most important aspects.
We re-arranged the discussion in three subsections as your critical comments.
Subsection 1 (lines 265–334): Loss-of-function variants of Epas1 in *S. galili* basalt populations
Subsection 2 (lines 335–399): Gain-of-function mutations of Epas1 in *E. baileyi*
Subsection 3 (lines 400–429): Negative regulation of the IFN pathway by the EPAS1 variants of both *S. galili* basalt populations and *E. baileyi*

Other individual points:
(1) line 114: how were the regulatory sequences identified? What makes them bona fide promotors?
To identify potential promoter regions, 3 kb upstream and 1 kb downstream from transcription start site (TSS) of *Epas*1 gene was analyzed with several promoter and transcription factor analysis tools available online (Castro-Mondragon et al, 2022). Sequence comparison of these populations revealed four variants in the 4-kb upstream regulatory region of the Epas1 gene. Nested PCR was used to clone a 3-kb and a 1.2-kb upstream region fragment of Epas1 from *S.galili.* Basalt and Chalk in Israel, respectively (Fig. S1A, B).

We have added these descriptions to the Results section. Additionally, we have included a 'Bioinformatics' subsection in the **Materials and Methods** section to provide a more detailed description."

Castro-Mondragon JA, Riudavets-Puig R, Rauluseviciute I, Lemma RB, Turchi L, Blanc-Mathieu R, Lucas J, Boddie P, Khan A, Pérez NM, Fornes O, Leung TY, Aguirre A, Hammal F, Schmelter D, Baranasic D, Ballester B, Sandelin A, Lenhard B, Vandepoele K, Wasserman WW, Parcy F, Mathelier A (2022) JASPAR 2022: the 9th release of the open-access database of transcription factor binding profiles. *Nucleic Acids Res* **50:** D165-D173

(2) Fig 1A: at the promotor variant sites, the allele frequency differences between the basalt and chalk populations of Sga are seemingly based on very few individuals (n=10). Do the observed differences excess genome-wide divergence? How can you make sure the variants are population-diagnostic? Also state the limitation that there is currently no evidence linking the regulatory sequence variants to reproductive isolation, speciation or fitness differences between populations.

"The allele frequency differences between the basalt and chalk populations of *S.galili.* are based on 10 individuals each. Although we are not claiming the variants to be population-diagnostic, -2023 site showed significant differentiation between basalt and chalk populations, with the AA genotype at 0.4 in chalk population and fixed (1.00) in basalt population. The same tendency was found in -1810 site, with the TT. genotype being 0.4 in chalk population and fixed (1.00) in basalt population. Larger sample size would strengthen the statistical power of the variation, but collecting additional individuals from the wild has proven to be logistically challenging. To assess the potential link between the regulatory sequence variants to hypoxia fitness differences of the two populations, EMSA, Luciferase reporter assay and RNA-seq were utilized to evaluate and clarify the functional relevance."

"Our results do not indicate that the variations directly drive the separation between the chalk and basalt populations, as there has been no evidence linking these variations to reproductive isolation and hence speciation. However, the regulatory outcome by these variations may benefit their adaptation." We have included and highlighted the above two paragraphs in Blue in MS.

• The effects of EPAS1 gene promotor variants have been studied in heterologous cells (HEK293T). Is there possibility to study transcription factor binding via ChiP-Seq in native Spalax tissue? If not, a sentence describing this limitation should be added.

We have added the following sentences to Discussion:

"We realize that a ChIP-seq in native Spalax tissues would be the best proof of the mechanisms. However, ChIP-grade antibody against Spalax EPAS1 protein is not currently available. We therefore performed our experiments in HEK293T cells to demonstrate the general regulatory mechanisms."

• Fig 2A, qRT-PCR: why was actin chosen as reference gene? Is stable expression of actin demonstrated? Lack of variants within primer binding sites verified (this could heavily influence quantification)? How many animals/biological replicates were used? (give number in legend)

ACTB is one of the most commonly used internal controls in mammalian qRT-PCR. To assess its suitability under our experimental conditions, we also evaluated several additional candidate reference genes, including 18S, GAPDH, and TBP, showing similar trends. Among these candidates, ACTB showed the most stable expression across the samples examined. Melting curve analysis and agarose gel electrophoresis further

supported the specificity of the amplification, with no evidence of primer-dimer formation or non-specific products. The number of biological replicates (n = 6 per condition, each derived from an independent animal) was originally provided in the Methods section, and we have now added this information to the legend of Fig. 2A as requested.

• lines 178-184: the experiment is not explained understandably. RNA-Seq ! was performed on exactly what? What are "low- and high dose" plasmids??
RNA-seq was performed on HEK293T cells transfected with either low- or high-amount of wild-type *S. galili* EPAS1 expression plasmids. The two plasmid doses were used to mimic the different EPAS1 expression levels observed between the basalt and chalk populations.

• RNA-Seq data should be uploaded to public database and acc. nos. given in the paper.
A Data Availability section has been added, indicating that the raw data are available from the corresponding author upon reasonable request."

• Stability of mutated EPAS1: there are no direct measurements of protein half-life or degradation kinetics. This limitation should be mentioned.
We agree with this point and have now addressed the limitation in the manuscript. Specifically, we have added the following sentence to the Discussion section:
"However, without direct measurements of EPAS1 protein half-life or degradation kinetics, the available information is insufficient to explain the stability of the mutated EPAS1."

• The causality between EPAS1 action and interferon signaling was inferred from a remote cell culture model. Is it possible to perform RNA-Seq on tissue from basalt and chalk specimen to measure interferon pathway gene activity in primary cells?
Tissue availability from *Spalax* is very limited, therefore RNAseq using tissues is not an option. Within the scope of the present work, we therefore used the different dose of HIF2a protein in HEK293T cells as an initial experimental system to mimic the varying EPAS1 expression levels observed in tissues from basalt and chalk. Using this system, we observed the enrichment of interferon pathway genes. We acknowledge that the underlying mechanism remains to be clarified. To address this, we plan to examine this question in primary *Spalax* fibroblasts in the future.

• Fig 5: why are mRNA and protein expression levels for the different collagen variants not measured side-by-side? (Col3a1 mRNA, Col1A1 protein) Is it safe that the anti-col1A1 antibodies robustly bind to the zokor protein?
Thank you for raising these important points. We would like to clarify as follows:

Side-by-side measurement of mRNA and protein for different collagen variants We did measure both *COL1A1* and *COL3A1* at the mRNA and protein levels. The transcriptomic data showed consistent**ly** decreased expression **of** *Col1a1* and *Col3a1* mRNA under 0.1% O2 hypoxia (Fig. 5E). **At the protein level,** we simultaneously tested COL1A1 and COL3A1 by WB. While COL1A1 was significantly decreased under 0.1% O2 in ***E.b*** fibroblasts (Fig. 5D), COL3A1 showed no significant alteration under same condition (**representative blot provided below**), For immunofluorescence, we focused on COL1A1 because it is a well-established fibrosis marker, and co-staining COL1A1 with F-actin was used to visualize matrix deposition.

Suitability of the anti-COL1A1 antibody for zokor protein Fibrillar collagens are highly conserved across mammals, particularly in the triple-helical domain. The antibody used in this study has been validated in multiple species, including human, mouse, and rat, and produced clear signals in both Western blotting and immunofluorescence in our samples, therefore we consider it suitable for zokor tissue under our experimental conditions.

Fibrillar collagens are highly conserved across mammals, particularly in the triple-helical domain. The anti-COL1A1 antibody (abcam, ab316222) used in this study has been validated in multiple species, including human, mouse, and rat. In our hands, it produced clear and specific signals in both Western blotting and immunofluorescence using zokor samples. The assay was carried out in strict accordance with the manufacturer's instructions. The concentration of our samples (1:500) was confirmed to fall within the assay's validated range. We have added the above clarifications to the revised manuscript as appropriate.

[Figure removed by editorial staff per authors' request].

• TGFbeta pathway involvement in zokor was not directly measured. This should be mentioned as limitation.

Thank you for this valuable suggestion. We agree that direct measurement of TGFβ pathway involvement in zokor was not performed in the current study, and we have now acknowledged this as a limitation in the revised manuscript. "Our interpretation of potential pathway involvement is derived from RNA-seq data showing differential expression of TGFβ-related genes; further experimental validation is required to confirm these findings."

• Evidence for an antifibrotic condition in zokor versus rat is limited: can you identify matching RNA-Seq data from rat to demonstrate species-specific and genome-wide response of fibrosis-associated genes?

We agree that direct comparison with rat RNA-seq data is not available. However, multiple published studies in mice and rats have shown that fibrotic stimulation consistently upregulates collagen genes, which are widely used as fibrosis markers. In our rat cell culture model, these markers were clearly increased under fibrotic conditions, whereas zokor cells did not show the same response. We have revised the manuscript to clarify this interpretation and explicitly acknowledge the lack of matched rat RNA-seq data as a limitation.

Additional points for revision:

• The text is not very smoothly written, also containing jumps of thoughts, making it cumbersome to read. Language editing is advised. In Results, each new section should shortly introduce the following line of experiments and why they were made...

Thank you for this helpful feedback. We have revised the manuscript throughout to improve readability and logical flow. Specifically, we have added short introductory sentences at the beginning of each subsection in the Results section to briefly explain the rationale for the following experiments. Language editing has also been applied. We believe the revised text is now substantially clearer and more coherent.

• Avoid jumping between gene designations EPAS1 and Hif2a.

Thank you for pointing this out. We apologize for the inconsistent use of gene designations. We have now carefully revised the entire manuscript to use the correct designations — **EPAS1** and **Hif2a** — consistently throughout, according to their respective species and context.

• Reference section: many citations are incomplete and require thorough editing. Inside the text there are wrong citation names, e.g. Katie O'Brien 2020 should be O'Brien and Murray.

Thank you. We have thoroughly edited the reference section to ensure all citations are complete and properly formatted. Regarding the specific example, the paper by O'Brien, Simonson, and Murray is now correctly cited in the text as (O'Brien et al., 2020). All other in-text citations have also been corrected. We apologize for these errors.

• For species abbreviations in text and figures, please stick to accepted rules (first letter of genus plus initial 2 letters of species designation. E. g. use Sga for Spalax galili). Avoid unnecessary and confusing punctuation like in "S.g." or "M.B.".

Thank you for this important suggestion. We have revised our species abbreviations throughout the text and figures to follow the accepted rules. Specifically, we now use ***S. galili*** for *Spalax galili* as in Nevo's paper (Nevo, 2013), ***E. baileyi*** for *Eospalax baileyi* (formerly *Myospalax baileyi*), and ***E. cansus*** for *Eospalax cansus* (formerly *Myospalax cansus*) as in An's paper (An et al, 2024). All confusing punctuation such as "S.g." or "M.B." has been removed. We believe this revision improves clarity and consistency with field standards.

An X, Mao LY, Wang YJ, Xu QQ, Liu X, Zhang SZ, Qiao ZL, Li BW, Li F, Kuang ZR, Wan N, Liang XL, Duan QJ, Feng ZL, Yang XJ, Liu SY, Nevo E, Liu JQ, Storz JF, Li KX (2024) Genomic structural variation is associated with hypoxia adaptation in high-altitude zokors. *Nat Ecol Evol*

Nevo E (2013) Stress, adaptation, and speciation in the evolution of the blind mole rat, Spalax, in Israel. *Mol Phylogenet Evol* **66:** 515-525

• line 39: "link transcriptional activity and the mRNA transcription" > ????

Thank you for pointing out this redundancy. We have revised the sentence to remove the awkward phrasing. The original "link transcriptional activity and the mRNA transcription" has been changed to **"linking transcriptional activity to interferons mRNA transcription"** for greater clarity and accuracy.

• line 68: delete "dramatic"

We have deleted it.

• line 76: higher than Mc, I guess?

"*E.baileyi* has **higher** red blood corpuscle counts and hemoglobin concentrations than that of pikas (*Ochotona curzniae*) and Sprague-Dawley rats" .

• line 80: use latin symbol for alpha throughout

Yes, we have revised it.

• line 89: any newer review available?

We have cited a newer review: The effect of HIF on metabolism and immunity…

• line 106: instead of "meanwhile" you may use "In parallel lines of investigation"

We have replaced "meanwhile" with "In parallel lines of investigation"

• lines 107-110 are not clearly phrased

We have revised these sentences and made it clearly as following:

"In parallel lines of investigation, four SNPs in the *Epas*1 regulatory region were found in two adjacent populations of *S. galili* in Israel, and these SNPs alter *Epas*1 gene transcription. These variants resulted in suppression of the interferon signaling pathway and fibrosis in *S.galili* Basalt population, *E.baileyi* and *E.cansu*s, which may contribute to the ecological adaptation of these species to their subterranean environments."

• line 132-133: why are bamboo rats introduced here? Define "R.p."

Thank you for noting this. Bamboo rats (*Rhizomys pruinosus*) belong to the same family (Spalacidae) as plateau zokors and Gansu zokors, and also carry the T189S and T480S mutations. We have now defined the species as ***R. pruinosus*** in the text and removed the undefined abbreviation "R.p."

• line 137 lacks introduction to the performed positive selection test applications. There is also nothing to be found in the MM section.

Thank you for noting this. We have now added the introduction and description of the positive selection test applications to both the Results section and the Materials and Methods section, providing the necessary context and methodological details.

• line 167: "variant of EPAS1 regulates promotor activity" >>>> really??

We have revised the sentence to more accurately reflect what was tested. The revised sentence now reads: "To explore how the variant of Epas1 regulates promoter activity, we tested the transcription of Epas1 in *S. galili* (Chalk and Basalt) using dual-luciferase reporter assays."

• line 167/168: I do not think the DLRA measured transcription of EPAS1

Thank you for catching this inaccuracy. We agree that dual-luciferase reporter assays measure promoter activity on a reporter gene, not direct transcription of the endogenous *Epas1* gene. We have revised the sentence accordingly: "To quantify the impact of regulatory variants, we measured the promoter activities of *Epas*1 on a reporter gene using dual-luciferase reporter assays."

• lines 174-175: what was done exactly? Description is unclear....

Thank you for pointing out the lack of clarity. We have revised the sentence to better describe exactly what was done in the EMSA experiments. The revised sentence now reads: " EMSA was performed using wild-type and variant-type biotin-labeled double-stranded probes. We found that the –2023A variant abolished the binding of cMyb in the upstream regulatory region of Epas1, while the –1810T variant increased the binding of HNF4G at the Epas1 regulatory region (Figs. 3B and S2A)." We believe this revision provides a clearer and more accurate description of the experimental approach and the key findings.

• line 261: you certainly mean "mutation hot spot"

Thank you for catching this error. You are correct — we meant "mutation hot spot." We have revised the sentence accordingly from: *"which mimics a human hot mutation in cancer"* to: *"which mimics a human mutation hot spot in cancer."* We appreciate your careful reading.

• lines 262-263: the sentence is messed up: variants in the regulatory region...regulate the expression of target genes....via multiple transcription factors (????)

Thank you for pointing out that the original sentence was unclear. We agree that the phrasing "via multiple transcription factors and transcription patterns" was vague and poorly constructed. We have revised the sentence to more accurately and clearly state our hypothesis: *"Therefore, we hypothesize that variants in the regulatory region of S. galili Epas1 affect its transcription by altering the binding of certain transcription factors, which may be involved in responding to fluctuating* O_2 *and* CO_2 *levels in subterranean caves."* We believe this revision eliminates the confusion and presents the hypothesis in a logical and precise manner.

June 11, 2026

RE: Life Science Alliance Manuscript #LSA-2026-03622-TR

Prof. Xue-Qun Chen
Zhejiang University
Neurobiology and Physiology
School of medicine, Research building C-507
West lake region
Hangzhou, Zhejiang 3100058
China

Dear Dr. Chen,

Thank you for submitting your revised manuscript entitled "Variants of EPAS1 Contribute to Hypoxia Adaptation in the Subterranean Rodents Eospalax and Spalax". Your revised manuscript was evaluated by both the original reviewers, whose comments are appended below. As you will note, both reviewers acknowledge your revised version and efforts to address their previous concerns. Reviewer 2 has sought additional clarifications and a minor concern around nomenclature. We encourage you to resolve all these concerns with appropriate edits in your manuscript. These are minimal edits and so we hope they will not take you much time.

In line with the reviewers' overall evaluation, we would be happy to publish your paper in Life Science Alliance pending resolution of the above minor points and final revisions necessary to meet our formatting guidelines. We request you to submit a revised manuscript document with all these changes highlighted along with a point-by-point response to the reviewer's comments.

MANUSCRIPT ORGANIZATION AND FORMATTING:

To avoid unnecessary delays in the acceptance and publication of your paper, please read the following information carefully. Full guidelines are available on our Instructions for Authors page, https://www.life-science-alliance.org/authors

Submission of a paper that does not conform to Life Science Alliance guidelines will delay the acceptance of your manuscript.

-As indicated by Reviewer 2, and in accordance with LSA guidelines on data availability (https://www.life-science-alliance.org/editorial-policies#data-sharing), we strongly encourage you to deposit your transcriptomics dataset to a recommended repository and include the name and accession ID in the section titled, 'Data Availability'.
-Please confirm that your ethical statement as reported in lines 441-444 applies to all experiments involving vertebrate animals in the study. Kindly move this statement to the start of the section titled, "Animals".
-In the legends, please provide a source for all the photographs used in Figures 1D and 1E.
-Please include molecular weight markers for blots shown in Figures 3, S1, and S2.
-Kindly label axes in Figures S2.
-Please include antibody concentrations to the details under Western blotting section, line 544.
-Please provide excitation/emission details for fluorescence microscopy. Related to this, kindly include scale bar size information in the legend for Figure 5F-H.
-Please upload your main and supplementary figures as single files.
-Please upload your supplementary tables separately.
-It is recommended to exclude figures from the manuscript text and upload them separately.
-Please add a Summary Blurb/Alternate Abstract and Keywords for your manuscript in our system.
-Please add the X and Bluesky handles of your host institute/organization, as well as your own, and/or one of the authors, in our system.
-Please add an Author Contributions section to your main manuscript text.
-The contributions selected for Ji-Zeng Du do not qualify them for authorship. Please either update the contributions in our system and in the Author Contributions section of the manuscript, or let us know if the author needs to be removed (and added eventually to the acknowledgment section).
-Please use the [10 author names et al.] format in your references (i.e., limit the author names to the first 10).
-Please add your main, supplementary figure, and table legends to the main manuscript text after the references section.
-Please add callouts for Figures 1E and S2C to your main manuscript text.
-Please be sure that the authorship listing and order is correct.

We encourage our authors to provide original source data, particularly uncropped/-processed electrophoretic blots and

spreadsheets for the main figures of the manuscript. If you would like to add source data, we would welcome one PDF/Excel-file per figure for this information. These files will be linked as supplementary "Source Data" files.

It is Life Science Alliance policy that if requested, original data images must be made available to the editors. Failure to provide original images upon request will result in unavoidable delays in publication. Please ensure that you have access to all original data images prior to final submission.

If you are planning a press release on your work, please inform us immediately to allow informing our production team and scheduling a release date.

We welcome submissions of potential cover images for the issue of LSA in which your work would appear. If you have high quality images associated with this work, please feel free to email these, with a caption, to the journal office.

LSA encourages authors to provide a 30-60 second video where the study is briefly explained. These videos will be appear embedded with the manuscript online at Life Science Alliance, and on social media to promote the published paper and authors (for examples, see https://docs.google.com/document/d/1-UWCfbE4pGcDdcgzcmiuJl2XMBJnxKYeqRvLLrLSo8s/edit?usp=sharing). Corresponding or first-authors are welcome to submit the video. Please submit only one video per manuscript. The video can be emailed to contact@life-science-alliance.org

FINAL FILES:

To upload the final version of your manuscript, please log in to your account: https://lsa.msubmit.net/cgi-bin/main.plex
You will be guided to complete the submission of your revised manuscript and to fill in all necessary information. Please get in touch in case you do not know or remember your login name.

The following items are required for acceptance.

-- An editable version of the final text (.DOC or .DOCX) is needed for copyediting (no PDFs).

-- High-resolution figure, supplementary figure and video files uploaded as individual files: See our detailed guidelines for preparing your production-ready images, https://www.life-science-alliance.org/authors

-- Summary blurb (enter in submission system): A short text summarizing in a single sentence the study (max. 200 characters including spaces). This text is used in conjunction with the titles of papers, hence should be informative and complementary to the title. It should describe the context and significance of the findings for a general readership; it should be written in the present tense and refer to the work in the third person. Author names should not be mentioned.

The license to publish form must be signed before your manuscript can be sent to production. A link to the license to publish form will be available to the corresponding author only. Please take a moment to check your funder requirements.

Reviews, decision letters, and point-by-point responses associated with peer-review at Life Science Alliance will be published, alongside the manuscript. If you do want to opt out of having the reviewer reports and your point-by-point responses displayed, please let us know immediately.

Thank you for your attention to these final processing requirements. Please revise and format the manuscript and upload materials as soon as you are able.

Thank you for this interesting contribution to the literature. We look forward to publishing your paper in Life Science Alliance.

Sincerely,

Sarita Hebbar, PhD
Scientific Editor
Life Science Alliance
http://www.lsajournal.org

Reviewer #1 (Comments to the Authors (Required)):

I thank the authors for responding to my comments. In particular, I appreciate the information on the bioinformatics analyses and the authors' efforts to make figures more accessible to color-blind readers. I find that the manuscript is improved after this revision and I have only a few minor comments to make.

- Throughout the text, there is generally no space between the genus name and the species name. For example, "S.galili" should be "S. galili".

- The authors mention the following in their response: "There are 15 introns in Tibetans and S.galili Epas1, and one SNP in intron 1 and another SNP in intron 2 of Tibetans Epas1 were identified as significant SNPs that correlated with a lower-than-expected Hb concentration (Hanaoka et al, 2012)." However, in line 92 of the main text, they still mention "... regulatory promoter, intron...". At the very least, they could change "intron" to "certain introns" or "introns 1 and 2".

- The acronym "DEGs" first occurs in line 191, whereas it is not explained until line 505.

- In lines 568-569, the authors mention that they inferred NJ and ML trees with MEGA X and provided them as input to CodeML. Does this mean that the authors ran each analysis twice, once with the NJ tree and once with the ML tree? This is not clear anywhere, especially given that tables S1 and S2 do not report separate estimates for each of the two trees.

Finally, I would encourage authors to reconsider how they will make their RNA-seq data available, especially given Life Science Alliance's policy on data availability: "Datasets obtained by experimentation, computation or data mining, should be made freely available, without restriction."

Reviewer #2 (Comments to the Authors (Required)):

The authors have adequately adressed the points raised in my previous review. I therefore recommend acceptance.

Response for #LSA-2026-03622-TR:

Dear Editor,
Thank you for your efforts and guidance. We have answered each reviewer's question in blue and revised the manuscript accordingly. We hope that the current version is much improved.
In addition, we have resolved every question you raised in your email as follows:

Please confirm that your ethical statement as reported in lines 441-444 applies to all experiments involving vertebrate animals in the study. Kindly move this statement to the start of the section titled, "Animals".
Done.
-In the legends, please provide a source for all the photographs used in Figures 1D and 1E.
Yes.
-Please include molecular weight markers for blots shown in Figures 3, S1, and S2. **included.**
-Kindly label axes in Figures S2.
Added.
-Please include antibody concentrations to the details under Western blotting section, line 544.
-Please provide excitation/emission details for fluorescence microscopy. Related to this, kindly include scale bar size information in the legend for Figure 5F-H. **provided**
-Please add an Author Contributions section to your main manuscript text. **Added**
-The contributions selected for Ji-Zeng Du do not qualify them for authorship. **finished**
-Please use the [10 author names et al.] format in your references (i.e., limit the author names to the first 10). **Done.**
-Please add callouts for Figures 1E and S2C to your main manuscript text. **Yes.**
-Please be sure that the authorship listing and order is correct. **Sure.**

Reviewer #1 (Comments to the Authors (Required)):

I thank the authors for responding to my comments. In particular, I appreciate the information on the bioinformatics analyses and the authors' efforts to make figures more accessible to color-blind readers. I find that the manuscript is improved after this revision and I have only a few minor comments to make.

- Throughout the text, there is generally no space between the genus name and the species name. For example, "S.galili" should be "S. galili".
Thank you for pointing this out. We have added a space between the genus name and the species name throughout the manuscript.

- The authors mention the following in their response: "There are 15 introns in Tibetans and S.galili Epas1, and one SNP in intron 1 and another SNP in intron 2 of Tibetans Epas1 were identified as significant SNPs that correlated with a lower-than-expected Hb concentration (Hanaoka et al, 2012)." However, in line 92 of the main text, they still mention "... regulatory promoter, intron...". At the very least, they could change "intron" to "certain introns" or "introns 1

and 2".

We apologize for the oversight. We have now changed "intron" to "certain introns" in line 92 of the revised manuscript.

- The acronym "DEGs" first occurs in line 191, whereas it is not explained until line 505.

We have now explained the acronym upon first use in line 191 as "differentially expressed genes (DEGs)".

- In lines 568-569, the authors mention that they inferred NJ and ML trees with MEGA X and provided them as input to CodeML. Does this mean that the authors ran each analysis twice, once with the NJ tree and once with the ML tree? This is not clear anywhere, especially given that tables S1 and S2 do not report separate estimates for each of the two trees.

We only used the NJ tree for the CodeML analysis, because the branching patterns of the NJ and ML trees were essentially similar. The sentences in lines 558-559 was corrected as follows:

Trees constructed using MEGA X (Kumar et al, 2018) with NJ method were used as input for generating the CodeML site models of PAML.

Finally, I would encourage authors to reconsider how they will make their RNA-seq data available, especially given Life Science Alliance's policy on data availability: "Datasets obtained by experimentation, computation or data mining, should be made freely available, without restriction."

We understand the journal's policy on data availability. Our RNA-seq data have been made available under the name and ID number of the related experiments.

Reviewer #2 (Comments to the Authors (Required)):

The authors have adequately adressed the points raised in my previous review. I therefore recommend acceptance.

June 26, 2026

RE: Life Science Alliance Manuscript #LSA-2026-03622-TRR

Prof. Xue-Qun Chen
Zhejiang University
Neurobiology and Physiology
School of medicine, Research building C-507
West lake region
Hangzhou, Zhejiang 3100058
China

Dear Dr. Chen,

Thank you for submitting your Research Article entitled "Variants of EPAS1 Contribute to Hypoxia Adaptation in the Subterranean Rodents Eospalax and Spalax". It is a pleasure to let you know that your manuscript is now accepted for publication in Life Science Alliance. Congratulations on this interesting work.

The final published version of your manuscript will be deposited by us to PubMed Central upon publication.

Your manuscript will now progress through copyediting and proofing. It is journal policy that authors provide original data upon request.

At the proofing stage, we ask you to complete the statement on data availability and suggest you phrase it as follows, "All data needed to evaluate the conclusions in the paper are present in the paper and/or the Supplementary Materials. RNA-seq raw data for the all the datasets have been submitted as raw sequence reads to NCBI and will be available at a later date."

Reviews, decision letters, and point-by-point responses associated with peer-review at Life Science Alliance will be published, alongside the manuscript. If you do want to opt out of having the reviewer reports and your point-by-point responses displayed, please let us know immediately.

IMPORTANT: If you will be unreachable at any time, please provide us with the email address of an alternate author. Failure to respond to routine queries may lead to unavoidable delays in publication.

Scheduling details will be available from our production department. You will receive proofs shortly before the publication date. Only essential corrections can be made at the proof stage so if there are any minor final changes you wish to make to the manuscript, please let the journal office know now.

DISTRIBUTION OF MATERIALS:
Authors are required to distribute freely any materials used in experiments published in Life Science Alliance. Authors are encouraged to deposit materials used in their studies to the appropriate repositories for distribution to researchers.

You can contact the journal office with any questions, contact@life-science-alliance.org

Again, congratulations on a very nice paper. I hope you found the review process to be constructive and are pleased with how the manuscript was handled editorially. We look forward to future exciting submissions from your lab.

Sincerely,

Sarita Hebbar, PhD
Scientific Editor
Life Science Alliance
http://www.lsajournal.org
